# Metabolism leaves its mark on the powerhouse: recent progress in post-translational modifications of lysine in mitochondria

**DOI:** 10.3389/fphys.2014.00301

**Published:** 2014-09-02

**Authors:** Kyriakos N. Papanicolaou, Brian O'Rourke, D. Brian Foster

**Affiliations:** Division of Cardiology, Department of Medicine, The Johns Hopkins University School of MedicineBaltimore, MD, USA

**Keywords:** acetylation, succinylation, malonylation, glutarylation, heart, sirtuin, Sirt3, Sirt5

## Abstract

Lysine modifications have been studied extensively in the nucleus, where they play pivotal roles in gene regulation and constitute one of the pillars of epigenetics. In the cytoplasm, they are critical to proteostasis. However, in the last decade we have also witnessed the emergence of mitochondria as a prime locus for post-translational modification (PTM) of lysine thanks, in large measure, to evolving proteomic techniques. Here, we review recent work on evolving set of PTM that arise from the direct reaction of lysine residues with energized metabolic thioester-coenzyme A intermediates, including acetylation, succinylation, malonylation, and glutarylation. We highlight the evolutionary conservation, kinetics, stoichiometry, and cross-talk between members of this emerging family of PTMs. We examine the impact on target protein function and regulation by mitochondrial sirtuins. Finally, we spotlight work in the heart and cardiac mitochondria, and consider the roles acetylation and other newly-found modifications may play in heart disease.

## Introduction

Though mitochondria are the primary site for ATP production, they have also emerged as hubs of control within a cell. What fuel source to use, whether to divide or fuse, to live or die—these are only a few of the decisions made at the level of mitochondria that require the integration of signals about nutrient availability, redox status, and overall health of the cell. Defining these signals is key to understanding mitochondrial function and designing therapies to minimize mitochondrial dysfunction during disease. Evidence indicates that mitochondrial processes are subject to modulation by a number of post-translational modifications (PTM), including phosphorylation of serines, threonines, and tyrosines, as well as through the redox-induced modification of reactive cysteines to yield glutathionylated and S-nitrosylated proteins (reviewed in Foster et al., 2009; O'Rourke et al., 2011; Mailloux et al., 2014).

In this review, we consider the PTM of lysine residues, concentrating primarily on those found in the mitochondrion. What has become clear over the last few years is that there is an emerging class of lysine modifications that stem from the reaction of proteins with thioester-CoenzymeA (CoA) byproducts of metabolism. We begin by surveying the field of mitochondrial lysine acetylation, before introducing more recently discovered PTMs, including succinylation, malonylation, and glutarylation. In each case, we address some key questions. What protein substrates are modified and how is function affected? Are these PTMs modulated by enzymes and, if so, what factors govern their activity? How dynamic are these processes and what is their stoichiometry? The answers to these questions will help shape our perception of these new PTMs, i.e., do they constitute a novel form of signaling, or is their accumulation simply a form of “metabolic stress” to be minimized? Finally, where applicable, we spotlight recent work in cardiac mitochondria, and examine the role of these PTMs in models of heart disease.

## Lysine acetylation

Biological acetylation of histones was first reported by Allfrey et al. (1964), though initial measurements primarily captured N-terminal acetylation, i.e., the reaction with the α-amino group of the mature histone's first amino acid. It was not until 1968 that investigators realized there was a second, more labile, population of acetylated residues in histones, labeled at the ε-amino group of internal lysine residues (Gershey et al., 1968). The transfer of an acetyl group from acetyl-CoA to the ε-amino group neutralizes lysine's positive charge. In the nucleus, this is a critical step in gene regulation, and is acutely regulated by the specific action of enzymes: the histone acetyl transferases (HATs) and deacetylases (HDACs). In the mid-2000's, advances in mass spectrometry coupled with the development of acetylation specific antibodies greatly expanded the catalog of lysine acetylated proteins beyond the nucleus, and mitochondrial proteins figure prominently. Lysine acetylomes have now been compiled from bacteria and yeast to fruit flies (Zhang et al., 2009; Weinert et al., 2011; Henriksen et al., 2012) and mammals. Given the prokaryotic ancestry of mitochondria, we first consider evidence for acetylation in bacteria.

### Protein lysine acetylation in prokaryotes

In prokaryotes, acetylation has emerged as a central regulatory mechanism that coordinates metabolism in response to changes in nutrient status (Wang et al., 2010). In *S.enterica*, 191 proteins are reported to be lysine-acetylated and in *E.coli* this number currently reaches 349 (Wang et al., 2010; Zhang et al., 2013). Metabolic enzymes (e.g., aldolase, Pdh, and Mdh, to name a few) are common substrates for acetylation and ontologic classification shows that acetylation on metabolic regulators is more frequent than that observed, for example, on ribosomal proteins. The prokaryotic enzymes, Pat and CobB, regulate bacterial protein lysine acetylation and deacetylation respectively (Starai et al., 2002; Starai and Escalante-Semerena, 2004; Wang et al., 2010). The balance between acetylation/deacetylation is influenced by several conditions, including switching carbon sources (e.g., from glucose to citrate), growth phase, and availability of acetylphosphate (AcP) (Yu et al., 2008; Zhang et al., 2009; Wang et al., 2010; Weinert et al., 2013a). Consensus sites that may serve as hotspots for lysine acetylation/deacetylation have been examined and, although no distinct patterns emerge, having tyrosine or histidine at site +1 frequently correlates with acetylation (Zhang et al., 2009), though not in all bacterial species examined (Kim et al., 2013). Estimates of stoichiometry in *E.coli*, indicate that, for the bulk of acetylated lysines, occupancy is below 1% (Weinert et al., 2013a). This is consistent with more recent calculations, showing that the stoichiometry for 82% of acetylated sites was equal or below 10% and only a small fraction of sites (4%) exhibited stoichiometries larger than 20% (Baeza et al., 2014). High acetylation stoichiometries were found in proteins participating in metabolic pathways including the pentose phosphate pathway, glycolysis and the tricarboxylic acid (TCA) cycle (Baeza et al., 2014).

The observation that acetylation occupancy is low for the majority of sites prompts the question as to whether it serves as a regulatory PTM or is simply a non-specific epiphenomenon of metabolism. Ultimately, the answer is likely a bit of both, though it would be prudent not to summarily dismiss a site's regulatory potential on the basis of low stoichiometry alone. Though most would agree that high site stoichiometry is a promising sign of its potential for protein regulation, it is no guarantee. Conversely, low PTM stoichiometry can have profound regulatory consequences if the target performs a key step in feed-forward processes such as enzymatic cascades or co-operative systems.

Regardless of stoichiometry, there are clearly examples of functional regulation by acetylation/deacetylation in prokaryotes. In *S.enterica* acetylation of GapA (prokaryotic homolog of Gapdh) stimulates glycolysis, while deacetylation reverses the equilibrium toward gluconeogenesis (Wang et al., 2010). This model illustrates one example where acetylation promotes the enzymatic activity of its substrate and feeds forward for the production of more acetyl-CoA through glycolysis. An example of feedback inhibition in *S.enterica* is the regulation of Acs (acetyl-CoA synthetase) by Pat and CobB. When acetyl-CoA is in excess, Acs is acetylated and deactivated by Pat and when glycolytic substrates become limited, CobB deacetylates Acs and activates the enzyme to synthesize acetyl-CoA from acetate (Thao and Escalante-Semerena, 2011). Another example in *E.coli* is the regulation of RNA polymerase (RNAPα) via acetylation at the C terminal domain (CTD) (Lima et al., 2012). With bacterial acetylomes expanding, we expect that more examples of protein regulation by acetylation will emerge thus improving our understanding of the biological significance of this modification.

### Protein lysine acetylation in yeast and flies

Acetylomes have been reported for model organisms such as *Saccharomyces cerevisiae* (Brewer's yeast) and *Drosophila melanogaster* (fruit fly), while for others, such as *Caenorhabditis elegans* (nematode worm) and *Danio rerio* (zebrafish), broad-scale proteomic assessments of lysine acetylation have yet to be described. Current studies have identified 959 or 1059 acetylated proteins in yeast (Henriksen et al., 2012; Weinert et al., 2013b) and 1013 acetylated proteins in the SL2 fruit fly cell line (Weinert et al., 2011). Proteins from every functional class are subject to acetylation (e.g., RNA processing, protein synthesis) while almost every enzyme participating in major metabolic pathways (e.g., glycolysis, gluconeogenesis) is acetylated in yeast (Henriksen et al., 2012). The evolutionary conservation of acetylated lysines is higher than non-acetylated lysines (Weinert et al., 2011; Henriksen et al., 2012) and sites of lysine acetylation are more tightly conserved than sites of phosphorylation (Weinert et al., 2011). Nevertheless, the stoichiometry of phosphorylation in yeast is significantly higher than acetylation, where the majority of acetylated sites have stoichiometries lower than 1% (Weinert et al., 2013b). The few exceptions with high acetylation stoichiometries are proteins already known to undergo acetylation by specific acetyl-transferases (largely related to acetylation in the nucleus). For other proteins, acetylation may occur as a low-frequency, non-enzymatic reaction driven by the concentration of acetyl-CoA (Weinert et al., 2013b). As discussed below, non-enzymatic modification is one of the proposed mechanisms underlying the acetylation of proteins in mitochondria (Newman et al., 2012; Wagner and Payne, 2013; Weinert et al., 2013b).

### Chemical and enzymatic control of mitochondrial acetylation/deacetylation in mammals

As in prokaryotes, lysine acetylation in mitochondria is tightly coupled to nutrient availability. The mechanism underlying acetylation, however, is still debated. It has been recently suggested that elevated *p*H in the mitochondrial matrix is favorable for the acetylation reaction (Wagner and Payne, 2013). Moreover, the mitochondrial matrix is where enzymes of the TCA cycle maintain high concentrations of acetyl-CoA. Together, these conditions would favor non-enzymatic formation of acetyl-lysine (Wagner and Payne, 2013). Additionally, Gcn5l1 is recently identified as a putative mitochondrial acetyltransferase in HepG2 cells (Scott et al., 2012). Gcn5l1, shares sequence similarity with bacterial acetyltransferases, localizes to mitochondria (both in the matrix and the IMS), and promotes mitochondrial protein acetylation in cells and in cell-free systems (Scott et al., 2012). Substrates of Gcn5l1 include electron transport chain (ETC) subunits Ndufa9 and Atp5a. It should be noted however that for full activity, Gcn5l1 requires additional mitochondrial factors and/or partners that are presently unknown. An intriguing observation in the two studies examining non-enzymatic acetylation or Gcn5l1-mediated acetylation is that mitochondrial proteins are more prone to acetylation when in their denatured state (Scott et al., 2012; Wagner and Payne, 2013).

By contrast to acetylation, there is no dearth of information regarding enzyme-driven mitochondrial deacetylation. Mammals host a family of seven genes called sirtuins 1 through 7 (Sirt1-7), due to their sequence homology to yeast silent information regulator 2 (Sir2p) (Haigis and Guarente, 2006). Three of the sirtuins (Sirt3, Sirt4 and Sirt5) are found in mitochondria (Onyango et al., 2002; Schwer et al., 2002; Michishita et al., 2005; Shi et al., 2005). The sirtuins use NAD^+^ co-substrate, which makes them particularly sensitive to fluctuations in metabolism. Notwithstanding their homology to yeast Sir2p, not all of the mitochondrial isoforms are equally effective as NAD-dependent deacetylases. Of the three mitochondrial sirtuins, Sirt3 appears to have the broadest deacetylase activity *in vivo* and will be discussed in subsequent sections (Lombard et al., 2007). Sirt4 is more widely recognized for its ADP-ribosyl-transferase activity (Haigis et al., 2006; Ahuja et al., 2007), although some suggest this may not be its major enzymatic role (Du et al., 2009). Sirt4 *does* exhibit some deacetylase activity *in vitro* (Rauh et al., 2013) and may have a restricted, but important, set of substrates *in vivo*. Recently, Sirt4 has been implicated in the coordination of fatty acid metabolism (Laurent et al., 2013a,b), modulation of ATP release from mitochondria (Ho et al., 2013) and tumor suppression by inhibiting glutamine metabolism (Csibi et al., 2013; Jeong et al., 2013). It is likely that as our view of the PTM landscape in mitochondria expands, additional substrates and/or activities of Sirt4 will be uncovered. This is exemplified by the case of Sirt5, a sirtuin with limited deacetylase activity, whose function has become clearer since the identification of malonylation and succinylation (more later).

### Harnessing proteomics to map pathways targeted by acetylation

The breadth of lysine acetylation on mitochondrial proteins was appreciated from the early proteome-wide studies that used cells such as HeLa, Jurkat, NIH/3T3, MV4-11 (an AML cell line) and A549 (an epithelial cell line) (Kim et al., 2006; Choudhary et al., 2009) and also mitochondria fractionated from mouse or human liver (Kim et al., 2006; Zhao et al., 2010). These studies are largely responsible for establishing the proteomic methodology used to identify acetylation sites. The workflow entails the enrichment of post-translationally modified peptides, first by precipitation of proteins (extracted from mitochondria, cells, or whole tissues), digestion with trypsin (and sometimes Lys-C endopeptidase from *Lysobacter enzymogenes*) and immunopurification of acetyl-lysine-bearing peptides using specific antibodies. Peptide capture is followed by chromatographic separation using high performance liquid chromatography and tandem mass spectrometry (MS/MS). The outcomes of such studies include the reporting of 133 acetylated proteins in mouse liver mitochondria (MLM) in 2006, 1395 acetylated proteins in Jurkat cells in 2009 and 1047 acetylated proteins in human liver in 2010 (Kim et al., 2006; Choudhary et al., 2009; Zhao et al., 2010). These methods have now been broadly applied and have proven integral to elucidating which biochemical pathways are subject to modulation by a variety of stimuli.

A central theme has been to examine precisely how lysine acetylation varies with nutrient availability and dietary composition. Examples include (i) fasting for 12 or 24 h (Kim et al., 2006; Hirschey et al., 2010; Rardin et al., 2013a), (ii) fasting and re-feeding (16 and 2 h respectively) (Still et al., 2013), (iii) a calorie restriction (CR) regimen (e.g., from 86.4 to 64.8 kcal/week for 2 or 3 months) (Hallows et al., 2011; Hebert et al., 2013), and (iv) high fat feeding (for 1, 7, or 13 weeks) (Hirschey et al., 2011). Changing the nutrition status elicits profound changes to the acetylation profile, but the outcome is often complex. For example, both high fat feeding and CR are reported to increase overall acetylation in liver mitochondria (Schwer et al., 2009; Hirschey et al., 2011; Hebert et al., 2013). A recent study using Sirt3^−/−^ mice identified 1757 acetylated sites in liver mitochondria as targets for deacetylation by Sirt3 in the context of CR (Hebert et al., 2013). Another study using the same mice identified 283 acetylated sites as targets for deacetylation by Sirt3 during a period of 24 h of fasting (Rardin et al., 2013a).

Global scale lysine acetylomes have also helped map which biochemical pathways are affected. Besides mouse liver, acetylomes are also reported for 16 unfractionated rat tissues including heart (1294 acetylated proteins), brain (1653 acetylated proteins) and brown adipose tissue (705 acetylated proteins) and also human skeletal muscle (941 acetylated proteins) (Lundby et al., 2012). Comparisons across tissues revealed that acetylation occurs on proteins known to be physiologically important for each tissue. For example, in heart and skeletal muscle, 80% of the proteins involved in muscle contraction were acetylated. Moreover, owing to their high content in mitochondria, brown fat, skeletal muscle and heart exhibited acetylation on the highly abundant proteins of the TCA cycle and the ETC (Lundby et al., 2012). In the liver, a tissue with a very versatile metabolism, acetylation is prominent on proteins involved in: (i) glycolysis/gluconeogenesis, (ii) fatty acid oxidation, (iii) the TCA cycle, (iv) the ETC and ATP synthase, (v) the catabolism of branched chain amino acids and the urea cycle, (vi) ketogenesis and even the malate shunt, peroxisomal FAO and acetate metabolism. Examples of acetylated enzymes are summarized in Table 1. This table (based mainly on studies in liver) illustrates some of the recent trends in the field, including efforts to identify regulatory sites and sites that are *bona fide* targets for deacetylation by Sirt3. Indeed, studies with knockout mice reveal the importance of Sirt3 activity in maintaining many lysines in their deacetylated state. Nevertheless, there remains a considerable fraction of lysines whose acetylation status varies independently of Sirt3 genotype, while other acetylation sites remain static (Hebert et al., 2013; Still et al., 2013).

**Table 1:**
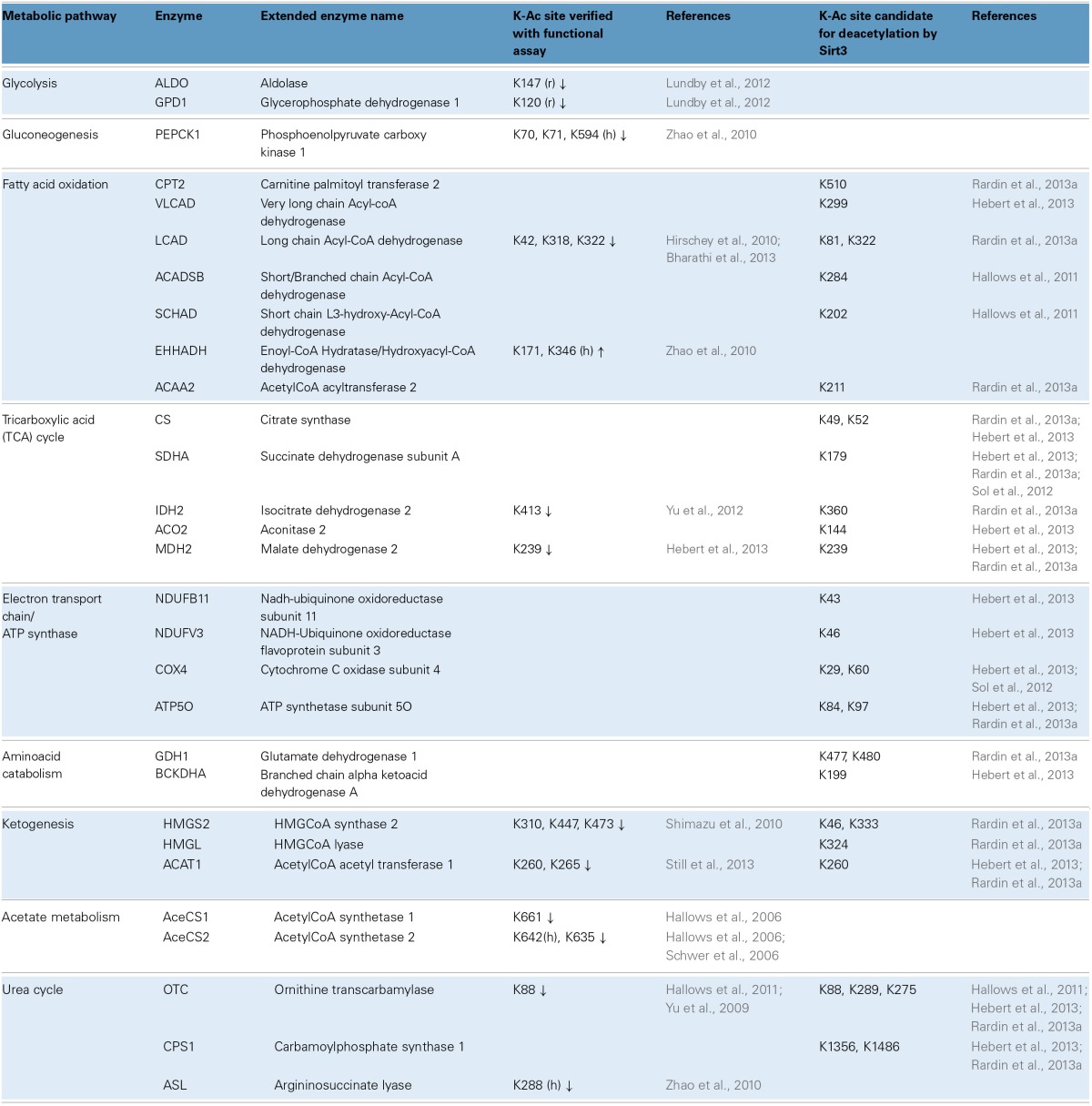
**Metabolic pathways and examples of acetylated enzymes/proteins identified by proteome-wide studies**.

*Arrows indicate whether the enzyme is activated (up) or inactivated by acetylation (down). Numbering of acetylation sites refers to mouse unless otherwise noted (h, human; r, rat)*.

Amid the flood of data, considerable effort is now aimed at assessing the functional consequences of target protein acetylation. Usually the candidate lysine (K) is mutated to either glutamine (Q) to mimic the charge-neutralizing effect of adding an acetyl group, or arginine (R), to render the site unacetylatable. These approaches have been used, for example, to show that acetylation of four lysine residues on cytoplasmic malate dehydrogenase (Mdh1) stimulate its enzymatic activity (Zhao et al., 2010). By contrast, acetylation of long chain acyl-CoA dehydrogenase (Lcad) at K42 is inhibitory (Hirschey et al., 2010), as is acetylation of ornithine transcarbamylase (Otc) at K88 (see also Table 1 for more examples). The mechanisms underlying enzyme inhibition seem to vary by protein. In certain cases acetylation at sites within or adjacent to the active site prevents catalytic steps such as substrate binding (Lundby et al., 2012; Bharathi et al., 2013; Hebert et al., 2013; Still et al., 2013). In others, such as Pepck1, acetylation is reported to promote degradation of the enzyme (Zhao et al., 2010; Jiang et al., 2011).

### Stoichiometry and dynamics of protein lysine acetylation in mammalian models

Proteomic studies have certainly revealed many mitochondrial acetyl-proteins, but is the extent or stoichiometry of acetylation sufficient to meaningfully affect their function? Fortunately, MS can provide clues on this front as well. For example, using enzyme immunoprecipitation, coupled with a quantitative proteomic strategy that used isobaric tags for relative and absolute quantification (iTRAQ)and the appropriate peptide standards, Zhao et al. estimated that the acetylation stoichiometry of malate dehydrogenase (Mdh2) and Enoyl-CoA Hydratase/Hydroxyacyl-CoA dehydrogenase (Ehhadh), in HEK293T cells ranges from 7% up to 47% depending on the specific site (Zhao et al., 2010). Moreover, acetylation levels increased substantially in response to treatment with trichostatin A and nicotinamide with profound effects on enzyme activity. It is important to note, that interventions such as knocking out genes or inducing acute and chronic changes in nutrition, can lead to changes in protein expression that can confound acetylation site quantification. In these cases, it is important to show that the abundance of the protein of interest does not vary significantly between groups (for example the abundance of Otc, Mdh2, Lcad, Sdha, and Hmgcs is similar between wild-type and Sirt3^−/−^ samples; Rardin et al., 2013a). Another common approach is to normalize the number of spectra obtained for an acetylated site after immunoenrichment to the total number of spectra obtained for that site in non-enriched samples. This approach was used to quantify changes in acetyl-lysine site occupancy under conditions of CR, fasting and refeeding or chronic overnutrition (Hebert et al., 2013; Still et al., 2013). Using normalization, it has also been estimated that the acetyl-lysine occupancy at K147 in aldolase reaches approximately 30% in the liver (Lundby et al., 2012).

A key corollary is: how long does it take to achieve appreciable changes in acetylation levels? Are the rates of acetylation and deacetylation comparable to those of phosphorylation and dephosphorylation? It has been suggested that factors influencing these dynamics may include intrinsic characteristics of the lysine residue being modified (i.e., position in the tertiary structure, making it more or less accessible for acetylation/deacetylation), or extrinsic factors such as the quantity and activity of deacetylases, or the availability of acetyl-CoA and matrix pH (Wagner and Payne, 2013; Pougovkina et al., 2014a). A survey of the literature reveals that in cell culture, widespread changes in acetylation are reported after 24 h of exposure to deacetylase inhibitors (Choudhary et al., 2009). Similarly, robust acetylation of Otc (see also Table 1) in cell culture is reported after 16 h of exposure to deacetylase inhibitor nicotinamide (Hallows et al., 2011). In liver mitochondria, changes in acetylation are detected as early as 2 h of acute re-feeding (Still et al., 2013). Fasting for 12 or 24 h also alters the acetylation pattern in MLM (Kim et al., 2006; Hirschey et al., 2010). Likewise acetylation is modulated by modest but long-term chronic changes in feeding. Chronic protocols such as 2 months of CR, 2.5 months of overnutrition or 3 months of high-fat feeding all yield changes in the acetylation patterns in liver mitochondrial proteins (Hirschey et al., 2011; Hebert et al., 2013; Still et al., 2013).

The deacetylase activity and protein levels of Sirt3 are regulated by nutrient availability. During a 16-h fast, Sirt3-dependent deacetylation occurs (Still et al., 2013), yet no changes in protein abundance are detected, suggesting that short-term fasting elicits Sirt3's enzymatic activity, likely through transient increase of NAD^+^ availability. However, after 24 h of fasting, Sirt3 protein levels significantly increase, through mechanisms requiring the transcriptional activator Pgc-1α (Hirschey et al., 2010). Furthermore, elevated protein levels of Sirt3 in the liver are also found after 2 or 3 months of CR (Hallows et al., 2011; Hebert et al., 2013). On the other hand, Sirt3 protein levels decline in liver mitochondria during the second or third month of high fat feeding (Hirschey et al., 2011). Thus, Sirt3 can respond to both acute and chronic changes in nutrition and its activation or inhibition can greatly influence the acetylation dynamics in mitochondria.

### Motifs in substrate sites that might signal acetylation and de-acetylation

Current efforts are also focused in the identification of consensus sites that are associated with higher frequency of acetylation and also sites that are subject to deacetylation by Sirt3 in mammalian cells and tissues. Early studies suggested that the presence of tyrosine (Y) or histidine (H) at position +1 is more preferentially identified on acetylated peptides (Kim et al., 2006; Choudhary et al., 2009). A more recent study however, did not identify significant trends for Y or H in position +1 of acetylated peptides in mitochondria (Lundby et al., 2012). In fact, among the different subcellular compartments examined, only nuclear proteins exhibited a strong acetylation motif, consistent with the presence of well-known acetyl-transferases in the nucleus. Therefore, with regard to mitochondria, a universal acetylation motif is not readily evident. On the other hand, efforts to identify sites that are preferentially de-acetylated by Sirt3 have yielded more consistent results. Thus, it appears that acetyl-K residues located on α-helical regions and flanked by other positively charged residues (i.e., K or R on positions +1 and +2) are more likely to be deacetylated by Sirt3 (Hebert et al., 2013; Rardin et al., 2013a).

## Protein acetylation in the heart and cardiac mitochondria

We now shift our attention to examine the roles of lysine acetylation in cardiac mitochondria and in systems that may be extrapolated to the cardiac domain. As in other organisms and organs, models ranging from SIRT ablation to CR, from cell biology to proteomics are providing insights that may one day be harnessed for therapeutic benefit.

### The role of Sirt3 in the heart and cardiac myocytes. candidate targets for deacetylation

Sirt3-deficient mice have been developed by several groups who, collectively have observed elevated acetylation on mitochondrial proteins in tissues lacking the gene (Lombard et al., 2007; Ahn et al., 2008; Yang et al., 2010; Fernandez-Marcos et al., 2012). Sirt3^−/−^ mice have baseline cardiac hypertrophy evident at 4 or 13 months of age (Sundaresan et al., 2009; Hafner et al., 2010), although data from others show no increase in the cardiac mass of Sirt3^−/−^ mice at 5 months of age (Someya et al., 2010). Sirt3 is induced in the heart by exercise or pathologic stimuli and is shown to protect against maladaptive cardiac remodeling and improve survival following pressure overload (Sundaresan et al., 2009; Hafner et al., 2010). It would be reasonable to presume that protection stems from deacetylation of mitochondrial substrates, yet initial evidence in cardiac cells has highlighted putative roles of Sirt3 outside mitochondria. Specifically, in neonatal rat ventricular myocytes (NRVMs), Sirt3 deacetylates nuclear protein Ku70, prevents mitochondrial translocation of the proapoptotic protein Bax, and enhances tolerance against H_2_O_2_ (Sundaresan et al., 2008). Moreover, Sirt3 deacetylates transcription factor Foxo3a, which increases the expression of manganese-dependent superoxide dismutase (MnSOD, Sod2) that, in turn, diminishes the accumulation of superoxide in mitochondria (Sundaresan et al., 2009). Data notwithstanding, it is still debatable whether Sirt3 is a *bona fide* regulator of Ku70 and Foxo3a, or whether enforced expression of Sirt3 accounts for the observed extra-mitochondrial activity (Bao et al., 2010). Furthermore, a quick survey in the acetylome database (http://cpr1.sund.ku.dk/cgi-bin/PTM.pl) failed to retrieve any acetylated peptides for Ku70 or Foxo3a in the heart, suggesting that the frequency of this modification might be below the detection limits of the MS/MS approach.

Acetylation of Sod2 is of considerable interest, since the enzyme is a critical component of mitochondrial antioxidant defenses and Sod2 deficiency exacerbates cardiac injury *in vivo* (Lebovitz et al., 1996). Although Sod2 acetylation in the heart has yet to be characterized fully, there is consensus among studies in liver, mouse embryo fibroblasts (MEFs), and HEK cells demonstrating that Sod2 activity is regulated via reversible acetylation and is controlled by Sirt3 (Qiu et al., 2010; Tao et al., 2010; Chen et al., 2011). For example, fasting for 36 h decreases the levels of acetylated Sod2 in the liver without reducing total protein levels and that regulation requires the presence of Sirt3 (Tao et al., 2010). Similarly, CR for 6 months (30% reduction in caloric intake) reduces the acetylation of Sod2 in the liver in a Sirt3-dependent fashion (Qiu et al., 2010). Moreover, Sirt3 is demonstrated to interact with and deacetylate Sod2 in MEFs and HEK293 cells, and in the absence of Sirt3, Sod2 exhibits increased acetylation and inhibited enzymatic activity *in vitro* (Qiu et al., 2010; Tao et al., 2010; Chen et al., 2011). Interestingly, each of these studies identified different lysine residues as the pivotal sites for regulation of Sod2 via reversible acetylation, including K53, K68 K89 and K122 (see also Table 2). Activation of Sod2 in the liver increases the ratio of [GSH:GSSG] (an index of antioxidant capacity) while decreasing indices of oxidative stress including hydroxynonenal, protein carbonylation and mitochondrial superoxide production (Qiu et al., 2010; Tao et al., 2010). Furthermore, Sod2 deacetylation curtails apoptosis in the liver and ameliorates cell death induced by paraquat (an inducer of oxidative stress). In MEFs, the expression of Sod2 deacetylation-mimetic mutants (i.e., K-to-R) abrogates mitochondrial superoxide production (Qiu et al., 2010; Tao et al., 2010). Thus, the activity of Sod2 depends on its acetylation state and it remains to be determined whether a similar mechanism is operable in the heart. Along these lines, it has been very recently reported that partial deficiency in Sirt3 is associated with lower Sod2 enzymatic activity in mouse hearts (Porter et al., 2014). These Sirt3 haploinsufficient hearts also demonstrated increased susceptibility to ischemia/reperfusion (I/R) injury (Porter et al., 2014).

**Table 2:**
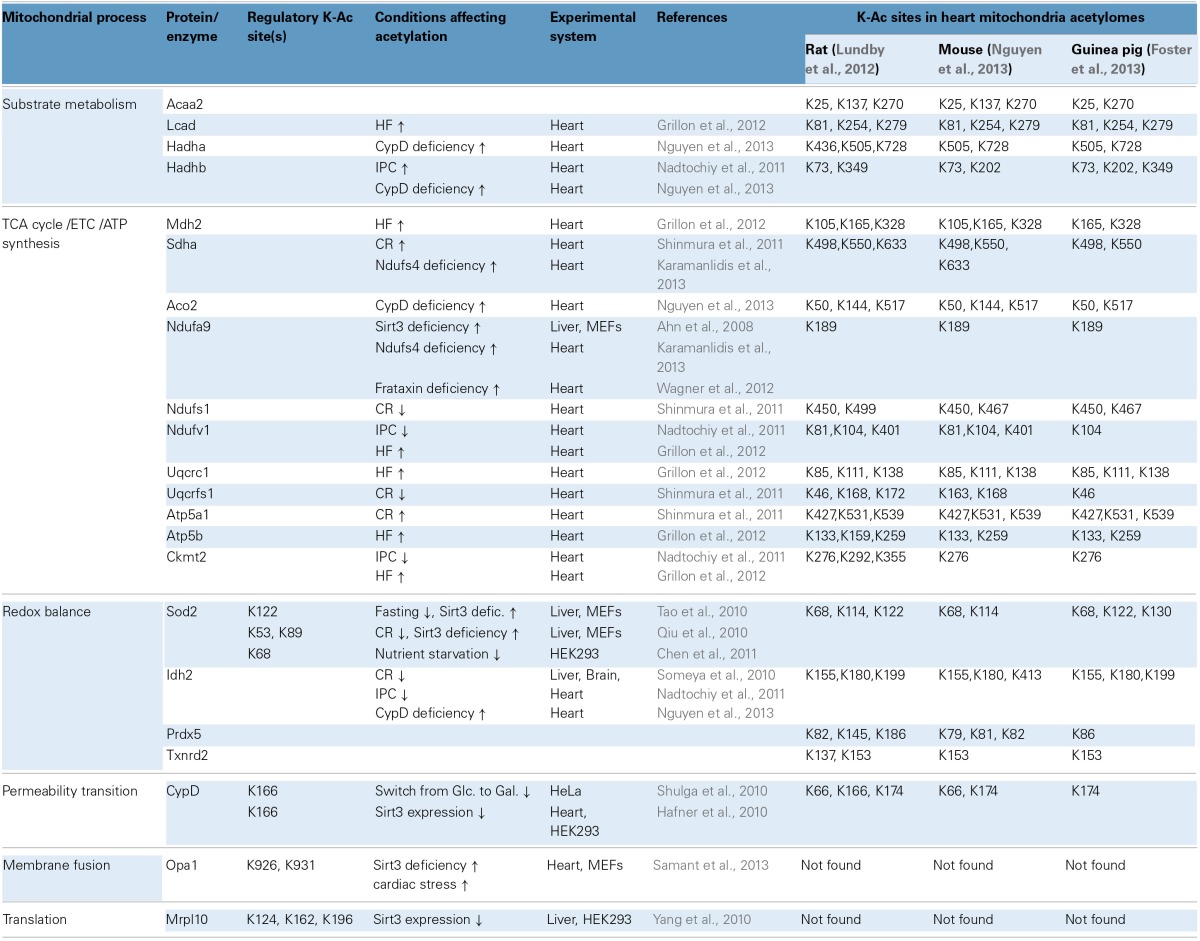
**Mitochondrial protein acetylation, regulatory sites and significance in cardiac physiology and pathophysiology**.

*IPC, ischemic preconditioning; CR, calorie restriction; HF, heart failure; Arrows indicate whether acetylation is increased (up) or decreased (down) by the different experimental conditions. K-Ac sites are reported according to mouse numbering; due to space limitations not all acetylated sites are shown, instead whenever possible, the conserved sites across species are shown; a blank cell indicates that no data are currently available*.

### Regulation of protein acetylation during calorie restriction in neurons and in heart

Like cardiac myocytes, neurons are post-mitotic cells with increased energy demands and heightened risk for oxidative damage. CR (25% reduction in calorie intake for 10 months) protected against neuronal death and hearing loss by activating antioxidant mechanisms in mice (Someya et al., 2010). A similar approach (40% reduction in calorie intake for 6 months) was also used to test the effects of CR in cardiac I/R injury in rats (Shinmura et al., 2011). In neurons, the protective effects of CR are mediated by increasing protein levels and activity of Sirt3 (Someya et al., 2010). In the heart, CR increases the overall mitochondrial sirtuin activity, although the protein levels of Sirt3 do not change significantly (Shinmura et al., 2011). In neurons, the anti-aging effects of CR implicate the deacetylation and activation of mitochondrial NADP-dependent isocitrate dehydrogenase 2 (Idh2; activation of this enzyme increases [NADPH:NADP+] ratio, which drives thiol-dependent antioxidant defenses). Likewise, CR up-regulates NADPH concentration and [GSH:GSSG] ratios in neuronal and liver mitochondria. Moreover, Idh2 maintains high levels of [NADPH] in HEK cells and protects against cell death induced by oxidants such as peroxide and menadione (Someya et al., 2010). In the case of heart, CR has protective effects in some but not all post-I/R indices of mitochondrial function. For example, oxygen consumption rates and mitochondrial H_2_O_2_ levels are largely unchanged by CR, although mitochondrial swelling due to Ca^2+^–overload is ameliorated (Shinmura et al., 2011). Acetylation of Idh2 was not studied in the CR-treated heart, but acetylation in a number of other proteins changed (see CR, Table 2). Notably, during this regimen, some mitochondrial proteins were deacetylated (Ndufs1, Uqcrfs1, and Sdha) whereas others became hyperacetylated (Atp5a1) (Shinmura et al., 2011). These targets offer starting points for further research into the cardioprotective mechanisms of CR.

### Regulation of cardiac protein acetylation in heart failure and ischemic preconditioning

Beyond cardiac I/R injury, mitochondrial protein acetylation has also been studied in the context of ischemic preconditioning (IPC), and experimental heart failure (HF). In the case of IPC, acetylation of mitochondrial proteins can be either upregulated or downregulated, a situation somewhat similar to that observed with CR-treated hearts. For example, the acetylation of Hadhb increases whereas that of Ndufv1 decreases during IPC (Nadtochiy et al., 2011) (see also IPC, Table 2). In HF however, there appears to be a more pronounced trend toward increased acetylation. In a study where rats developed HF at 7.5 or 18 months of age, it was determined that 41 or 66 cardiac proteins respectively exhibited increased acetylation (Grillon et al., 2012). Examples of acetylated proteins include Ndufv1, Uqcrc1, Mdh2, and Ckmt2 (see also Table 2) (Grillon et al., 2012). Increased mitochondrial protein acetylation is also shown in a murine model of diabetic cardiomyopathy (Vadvalkar et al., 2013). Provided that appropriate normalization is performed for changes in protein abundance, it can be suggested that HF progression is accompanied by increased mitochondrial protein acetylation. Notably, Sirt3 protein levels decrease in failing rat hearts or when H9C2 cardiomyocytes are subjected to oxidative stress (Grillon et al., 2012; Chen et al., 2013). Finally, notwithstanding the protein remodeling that might compromise mitochondrial function during HF progression, it has been shown that acute induction of acetylation chemically with acetic anhydride is sufficient to adversely impact mitochondrial function, specifically lowering state III respiration and elevating ROS production (Vadvalkar et al., 2013). Mitochondrial protein deacetylation might also be necessary for successful cardiac IPC, however the bulk of the current data center on important regulators of IPC in the cytosol. This is supported by the finding that a sirtuin inhibitor, splitomycin, negates the post-ischemic recovery of preconditioned hearts (Nadtochiy et al., 2011). Since splitomycin targets Sirt1 and is likely to block deacetylation of cytoplasmic proteins, it remains an open question whether deacetylation of mitochondrial proteins and the relevant sirtuin Sirt3 is required for effective IPC. It would therefore seem that accumulating evidence supports the argument that mitochondrial lysine acetylation in the heart is harmful. Alternatively, it has also been suggested that acetylcarnitine treatment may alleviate age-related mitochondrial dysfunction, in part, via acetylation and activation of mitochondrially-encoded gene expression (Rosca et al., 2009).

### Lysine acetylation on Cyclophilin D

Cyclophilin D (CypD) operates as a mitochondrial chaperone with *cis/trans* (C/T) isomerase activity but is best known as a modulator of acute necrotic cell death through the mitochondrial permeability transition (MPT). Studies in cancer cell lines and in HEK cells have suggested that acetylated CypD can more readily sensitize MPT (Hafner et al., 2010; Shulga et al., 2010). In HeLa cells growing in glucose, CypD is acetylated and has enhanced C/T isomerase activity whereas in cells growing in galactose, CypD is deacetylated by Sirt3 and its C/T isomerase activity declines (Shulga et al., 2010). The interaction between Sirt3 and CypD and the deacetylation of the latter is also documented in HEK cells (Hafner et al., 2010). K166 in CypD is identified as a critical site for deacetylation by Sirt3 (Table 2). This site is important for C/T isomerase activity and K166's acetylation may increase CypD's capacity to interact with other protein partners to induce MPT and reduce mitochondrial respiration rates (Shulga et al., 2010). Together, these studies provide an example where increased acetylation becomes detrimental, not by inhibiting enzymatic activity or protein complexes but by activating a protein to adversely affect mitochondrial functions. Nevertheless, it is increasingly recognized that not all CypD functions are detrimental to mitochondria. For example, CypD may be involved in the release of excess Ca^2+^ from mitochondria (Elrod et al., 2010).

### Other cardiac models associated with increased protein acetylation

**Figure 1:**
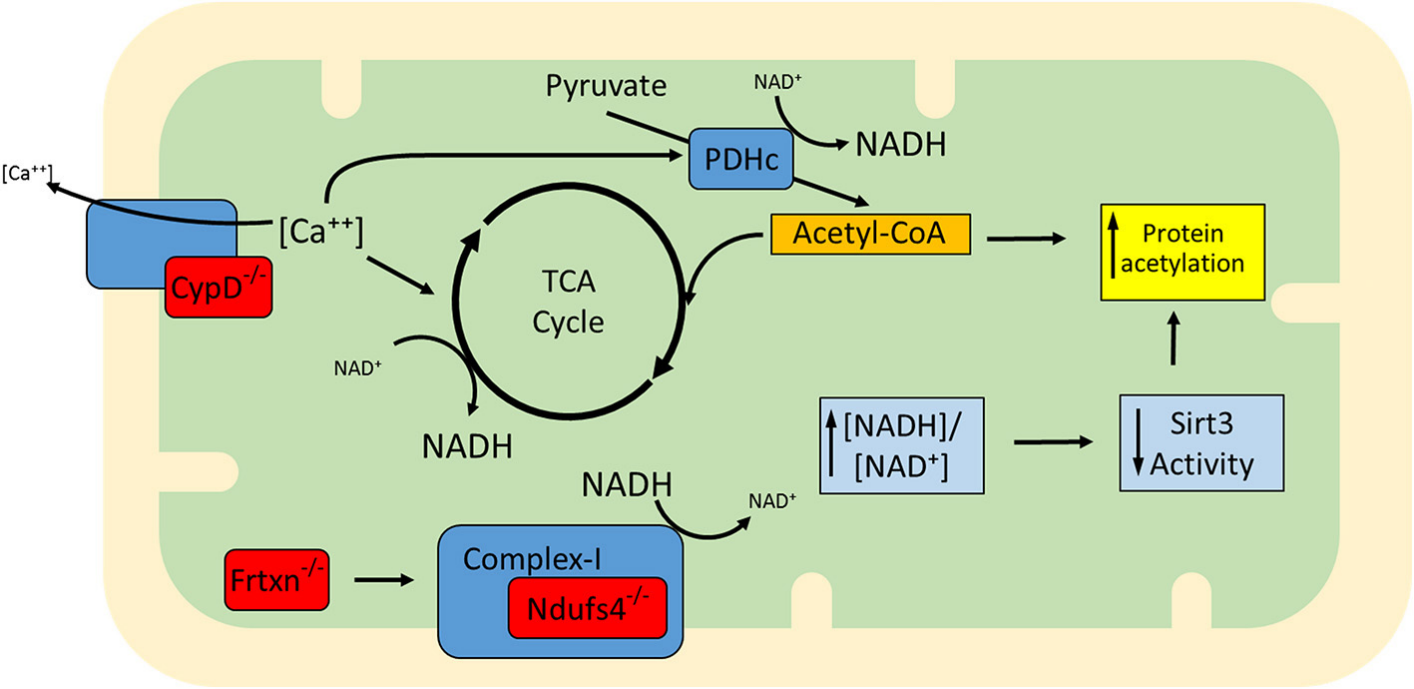
**Proposed model for the increased mitochondrial protein acetylation detected in the absence of Ndufs4, CypD, and frataxin (gene deficiencies shown in red)**. CypD facilitates Ca^++^ efflux and when absent, mitochondria exhibit higher than normal [Ca^++^]. This upregulates matrix dehydrogenases (e.g., TCA cycle dehydrogenases and PDH). Dehydrogenase activation enhances generation of NADH, altering the balance to higher ratios of [NADH:NAD^+^]. Complex I normally converts NADH back to NAD^+^ but in the absence of Ndufs4 this conversion is blocked. Defects in complex I activity may also arise in the absence of Fe-S cluster biogenesis, a mitochondrial process regulated by frataxin. Although described independently (Wagner et al., 2012; Karamanlidis et al., 2013; Nguyen et al., 2013), the three conditions are similar in that they exhibit an increase in protein acetylation which may be at least in part attributable to elevation of [NADH:NAD^+^] ratios and lowering of Sirt3 activity. It remains to be determined whether mechanisms other than Sirt3 inhibition (e.g., elevation of acetyl-CoA) also contribute to increased acetylation in these models.

Apart from ablation of Sirt3 (Lombard et al., 2007; Ahn et al., 2008; Fernandez-Marcos et al., 2012), a protein with documented deacetylase activity, other proteins have also been shown to have an impact on mitochondrial lysine acetylation levels, among them, Ndufs4, frataxin (frtxn), and CypD (Wagner et al., 2012; Karamanlidis et al., 2013; Nguyen et al., 2013). Ndufs4 is a subunit of complex I (NADH dehydrogenase) and its deletion in cardiac myocytes renders mitochondria deficient in complex I-driven respiration (Karamanlidis et al., 2013). Owing to complex I deficiency, the generation of NAD^+^ from NADH is blocked and the [NADH:NAD^+^] ratio is elevated. In frataxin deficiency, iron-sulfur cluster biogenesis and respiratory complex formation are impaired, again leading to elevated [NADH:NAD^+^] ratio (Wagner et al., 2012). In the case of CypD deficiency, mitochondria exhibit increased activity of matrix dehydrogenase complexes pyruvate dehydrogenase complex (PDHc) and KGDHc (Elrod et al., 2010) which can also elevate the [NADH:NAD^+^] ratio. Therefore, these gene deficiencies are characterized by increased [NADH:NAD^+^] ratios which would be expected to inhibit Sirt3, whose deacetylase activity is dependent on NAD+ (Figure 1) (Tanner et al., 2000). Consistently, mitochondria isolated from Ndufs4^−/−^, frtxn^−/−^, or CypD^−/−^ hearts have altered [NADH/NAD^+^] ratios and hyperacetylated proteins, despite unchanged Sirt3 protein levels (Wagner et al., 2012; Karamanlidis et al., 2013; Nguyen et al., 2013). It bears noting, however, that high NADH in these models could also lead to feedback inhibition of the TCA cycle and accumulation of acetyl-CoA. In turn, this might well contribute to hyperacetylation independently of Sirt activity.

Examples of proteins whose acetylation is increased in Ndufs4^−/−^, frtxn^−/−^, and CypD^−/−^ mitochondria are shown in Table 2. Sirt3 localizes to complex I where it deacetylates subunit Ndufa9, but fails to do so after treatment with rotenone (a complex I inhibitor) (Ahn et al., 2008). Furthermore, rotenone treatment increases mitochondrial protein acetylation to an extent similar to that observed when mitochondria are treated with nicotinamide (a sirtuin inhibitor) (Karamanlidis et al., 2013). These findings illustrate that even short term inhibition of complex I can increase protein acetylation in cardiac mitochondria. Likewise, with respect to CypD action, it would be interesting to test whether cyclosporine A (a CypD inhibitor) yields a similar result in protein acetylation. As for the downstream pathways that are affected in the three genetic models, Ndufs4^−/−^ mitochondria have unchanged [NADPH:NADP^+^] ratios, unchanged aconitase activity and unchanged acetylation levels of Sod2 all consistent with normal redox defense mechanisms (Karamanlidis et al., 2013). On the other hand CypD^−/−^ mitochondria have increased acetylation of Idh2 consistent with reduced capacity to counteract redox stress (Nguyen et al., 2013). Frtxn^−/−^ hearts have high levels of protein carbonylation, also suggesting reduced capacity to counteract redox stress (Wagner et al., 2012). Finally, at the level of cardiac function, Ndufs4^−/−^ hearts have normal function at baseline which declines following pressure overload (Karamanlidis et al., 2013). Decreased heart function following pressure overload is also reported for the case of CypD^−/−^ (Elrod et al., 2010), whereas frataxin deficiency is characterized by early stage cardiomyopathy (Puccio et al., 2001). Taken together, these models suggest that increased mitochondrial protein acetylation, caused by either altered [NADH:NAD^+^] and/or Acetyl-CoA levels, may be a common mechanism underlying the onset of cardiac dysfunction in response to pathological stressors (Figure 1).

### Cardiac acetyl-lysine proteome datasets

Though most global acetylome data sets have been obtained from liver, efforts to characterize the broader acetylation profile in the heart have begun. Following fractionation and mitochondrial purification, a gel-free proteomics approach identified about 130 acetylated proteins in the case of guinea pig heart (Foster et al., 2013). Similarly, about 200 acetylated proteins were identified in purified mouse heart mitochondria (Nguyen et al., 2013). Furthermore, proteomics in unfractionated rat hearts yielded about 245 acetylated proteins mapping to the mitochondrion (Lundby et al., 2012). Table 2 gives a snapshot of some of the mitochondrial substrates detected in the three species, the site(s) of acetylation for each substrate and highlights the functional categories where reversible acetylation might be important (i.e., substrate metabolism, TCA cycle, ETC, ATP synthesis, redox balance, and MPT). It becomes evident that as putative acetylation sites are uncovered by proteomics, there is an increasing demand to verify with functional assays the sites whose reversible acetylation has profound effects in protein function. No doubt, efforts will now turn to investigate how global-scale acetylation profiles are impacted in models of heart disease. Some evidence is already available from models discussed above (e.g., CR, IPC, HF), and large-scale quantitative proteomics will likely uncover new and important details on mitochondrial protein acetylation.

## Expanding the spectrum of protein lysine modifications. succinylation and the role of Sirt5

**Figure 2:**
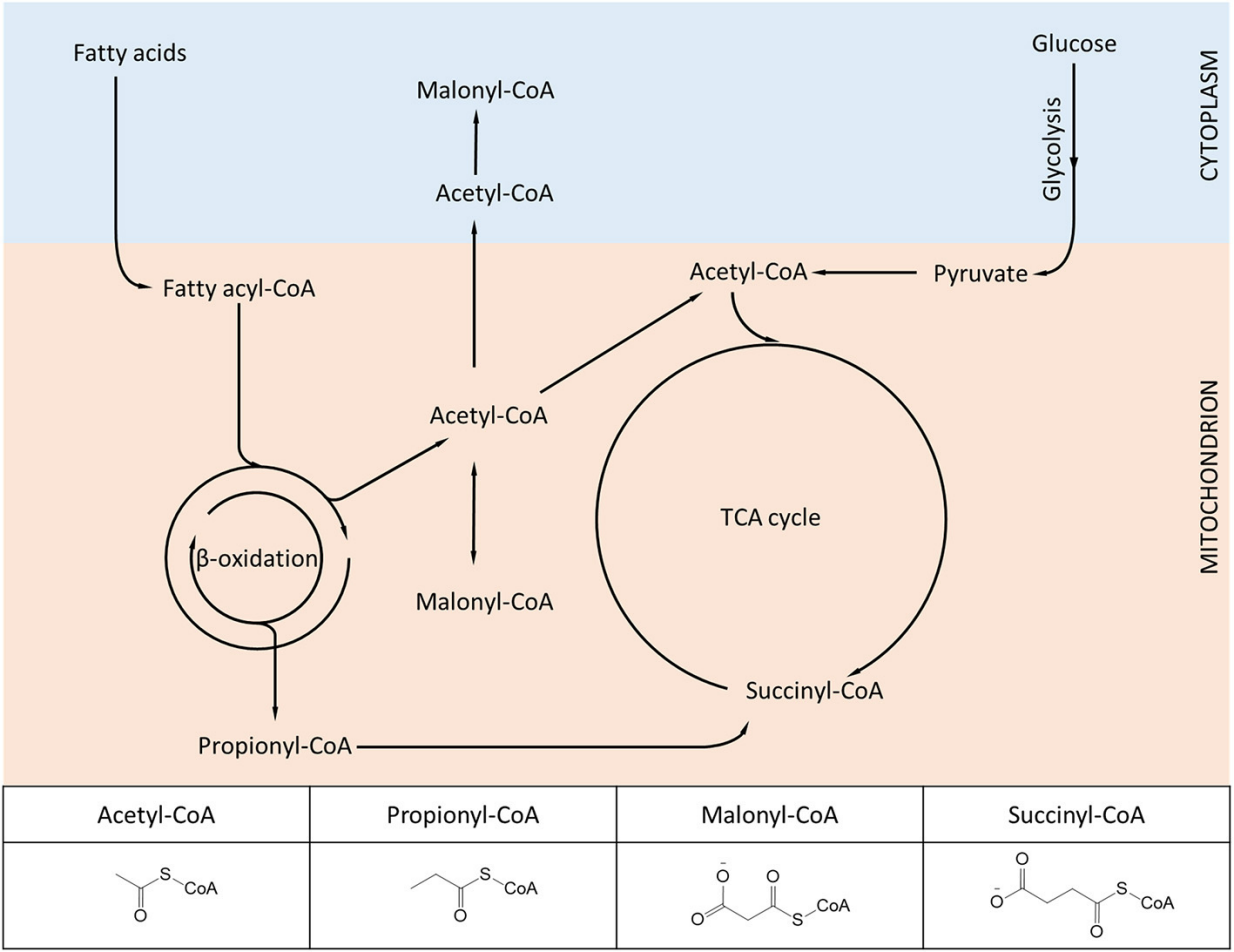
**Diagram showing metabolic intermediates produced in mitochondria and utilized in protein lysine modifications**. The inset table highlights the structural similarities and differences among the various acyl-CoA species. Lysine acetylation, propionylation, malonylation, and succinylation have been found to dynamically change in mammalian mitochondria, while other acylation variants (e.g., butyrylation, crotonylation) await further experimental characterization.

We have previously mentioned how lysine acetylation in mitochondria may be favored by the elevated pH of the matrix and the proximity of proteins to locally-generated acetyl-CoA. Yet acetyl-CoA is only one of several metabolite-CoA species generated in mitochondria (Figure 2), which given the same conditions, would also be expected to react with lysine. Again, the combination of technological advancements that led to characterization of the mitochondrial acetylome have been successfully applied for characterizing new modifications such as succinylation and malonylation.

### Lysine succinylation in bacteria and non-mammalian eukaryotic cells

Protein lysine succinylation (K-Su) results from the reaction of the dicarboxylate compound succinyl-CoA with lysine residues. The addition of a succinyl moiety to lysine adds a mass of 100 Da and imparts a negative charge at physiological pH. Using MS/MS, initial studies in *E.coli* identified 14 succinylated proteins including IcdA (homolog to mammalian Idh2), GapA (homolog to Gapdh) and several ribosome subunits and protein chaperones (homologs to mammalian Hsp60 and Hsp70) (Zhang et al., 2011). More recently, the tally has reached as high as 670 and 990 succinylated proteins in two recent studies (Colak et al., 2013; Weinert et al., 2013c). It is not yet clear what effects K-Su has on the function of protein substrates. To begin addressing these question, site-directed mutagenesis of K to E has been employed to mimic the creation of a K-Su site. In one experiment, a mutant IcdA K242E exhibited decreased rates of NADPH production *in vitro* suggesting that succinylation might be inhibitory for the dehydrogenase activity of IcdA (Zhang et al., 2011).

As in the case of acetylation, growth conditions influence the magnitude of succinylation in *E.coli*. Exposure to sodium succinate for 4 h increases the number of succinylated proteins (Zhang et al., 2011). Similarly, supplementation of minimal growth medium with either pyruvate, succinate or glucose, potently elevates global succinylation levels after 16 h (Colak et al., 2013). Estimates of succinylation stoichiometry on target proteins in *E.coli* are reported but they are only limited to a small number of proteins. The stoichiometry of protein succinylation in *E.coli* appears to range from 0.14 to 34%. For comparison, the stoichiometry of protein acetylation ranges from 0.22 to 73% in the same conditions (Colak et al., 2013). Succinylation (K-Su) appears to overlap considerably with acetylation (K-Ac). In *E.coli* grown in 0.8% glucose, K-Su and K-Ac sites overlapped by 39.3% (Colak et al., 2013), while in *E.coli* grown in 0.2% glucose, K-Su and K-Ac sites overlapped by 18.6% (Weinert et al., 2013c). It is not yet clear whether competition between acetyl- and succinyl-moieties for the same K site offers an additional regulatory level for controlling the activity of the protein substrate.

Bacteria contain a sirtuin-like protein (CobB) that functions as a protein deacetylase (Starai et al., 2002; Zhao et al., 2004; Weinert et al., 2013a). It was nevertheless hypothesized that CobB might have a broader specificity and function as a protein desuccinylase. Using wild-type and CobB-deficient bacteria, two studies reached different conclusions. In one study, the deletion of CobB did not induce considerable increases in global levels of protein succinylation (Weinert et al., 2013c). However, another study demonstrated that loss of CobB induced significant increases in global protein succinylation suggesting that CobB opposes protein succinylation (Colak et al., 2013). *In vitro* assays further confirmed CobB's ability to desuccinylate substrate peptides and highlighted residues within the protein that might be important for interacting with the K-Su substrate (Colak et al., 2013). It should be noted that the growth conditions in the two studies were different (i.e., in the presence of 0.2 or 0.8% glucose), which might in part be responsible for the discrepancies obtained regarding the desuccinylase activity of CobB. Once the role of CobB in desuccinylation is independently corroborated, it would be interesting to determine which K-Su substrate sites are subject to regulation by this enzyme and whether the enzyme can be regulated to preferentially distinguish between K-Su and K-Ac sites.

In addition to prokaryotic cells, lysine succinylation was identified in yeast (*S.cerevisiae*) and SL2 cells (*D.melanogaster*) (Zhang et al., 2011; Xie et al., 2012; Weinert et al., 2013c), suggesting that this PTM is evolutionarily conserved. A proteome-wide study in yeast identified 474 succinylated proteins (Weinert et al., 2013c). Notwithstanding that succinyl-CoA, the primary metabolite required for succinylation, is produced inside mitochondria (Figure 2) only a small fraction of proteins identified in the yeast succinylome (61 out of 474) map to mitochondria, while the majority are cytoplasmic and nuclear (Xie et al., 2012; Weinert et al., 2013c). Altering the availability of succinyl-CoA (by genetically ablating specific enzymes of the TCA cycle) affects the global succinylation pattern in yeast, suggesting that succinyl-CoA used for succinylation comes from mitochondria (Weinert et al., 2013c). Nevertheless, it is not yet clear how succinyl-CoA is exported to the cytoplasm. As in prokaryotic cells, K-Su and K-Ac sites tend to overlap, although in the case of yeast this is less pronounced (~16%) (Weinert et al., 2013c). While absolute stoichiometries of protein succinylation remain to be determined, the current evidence suggests that for most yeast proteins this is low, or at least much lower than the stoichiometries of phosphorylation. This evidence suggests that lysine succinylation in yeast operates as a low-grade PTM, although it might prove important for protein regulation under conditions that have yet to be identified. Another open question is whether yeast protein succinylation is subject to regulation by *Sir2*.

### Regulation of protein lysine succinylation by Sirt5

**Figure 3:**
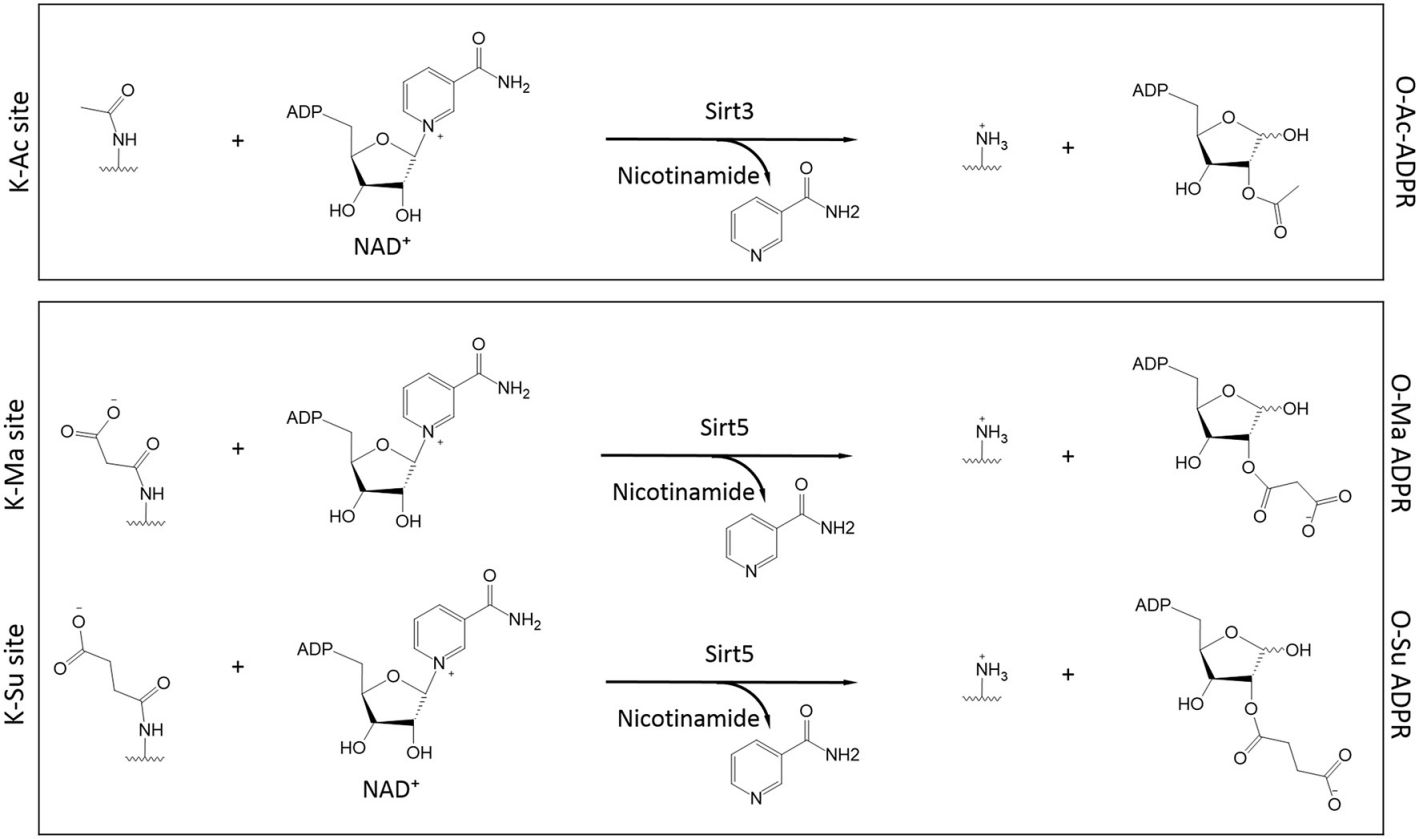
**Role of Sirt3 and Sirt5 in the catalysis of protein deacylation in the presence of NAD^+^**. Sirt3 has specificity for acetylated lysines (K-Ac) whereas Sirt5 has broader specificity and it can accommodate malonylated and succinylated substrates (K-Ma and K-Su respectively). In all cases, NAD^+^ is consumed to release nicotinamide while the ADP-Ribose (ADPR) moiety serves as the final acceptor of the acyl group (O-Ac-, O-Ma-, and O-Su-ADPR respectively). The acyl-modified and de-modified site is also shown.

Almost simultaneously, two groups made the seminal discovery that Sirt5, a mitochondrial sirtuin with only marginal deacetylase activity, had the ability to function as a *desuccinylase* (Du et al., 2011; Peng et al., 2011). Using a thin layer chromatography assay to monitor conversion of radiolabeled NAD^+^ to acyl-ADP-ribose, a hallmark of sirtuin activity, it was shown that high purity SIRT5 displayed activity toward chemically synthesized succinyl-lysine peptides (Du et al., 2011). Desuccinylation by Sirt5 was confirmed by HPLC-MS or fluorescent techniques and Sirt5's activity was sensitive to inhibition by nicotinamide (Du et al., 2011; Peng et al., 2011). A crystal structure of Sirt5, in complex with a succinylated peptide, indicated that residues Y102 and R105 are important for interactions with the negatively-charged carboxyl group of the succinylated lysine (Du et al., 2011). Furthermore, R105 is suggested to confer increased sensitivity to inhibition by nicotinamide (Fischer et al., 2012). Another Sirt5 residue, H158, is important for general sirtuin catalytic activity (Schwer et al., 2002; Nakagawa et al., 2009) and, consistently, a mutant H158Y exhibits no desuccinylase activity (Du et al., 2011; Peng et al., 2011). By immobilizing Sirt5 on a column and subjecting a bovine liver mitochondrial extract to affinity purification, the first substrates of Sirt5 were identified, including Got2 (glutamate oxaloacetate transaminase 2) and Hmgcs2 (HMG-CoA synthetase 2) (Du et al., 2011). The finding that Sirt5 operates as a protein lysine desuccinylase was instrumental in characterizing the functions and regulation of this PTM, as discussed in the section below. Of note, Sirt5 exhibits broader specificity for modified lysines and in addition to desuccinylation, it can also perform demalonylation and deglutarylation (Du et al., 2011; Peng et al., 2011; Tan et al., 2014) (Figure 3).

### Characterizing protein lysine succinylation in mammalian experimental systems

The study of succinylation in mammalian cells encompasses HeLa, HepG2, HEK293, and wild-type or Sirt5-deficient MEFs (Peng et al., 2011; Park et al., 2013; Rardin et al., 2013b; Weinert et al., 2013c). Among tissues, succinylation has been studied primarily in the liver (Du et al., 2011; Peng et al., 2011; Park et al., 2013; Rardin et al., 2013b; Weinert et al., 2013c; Yu et al., 2013; Buler et al., 2014) and skeletal muscle (Rardin et al., 2013b; Yu et al., 2013). Sirt5^−/−^ mice have been used in several studies (Lombard et al., 2007; Du et al., 2011; Peng et al., 2011; Park et al., 2013; Rardin et al., 2013b; Yu et al., 2013) and in many cases mitochondria have been the focal point (Fritz et al., 2013; Park et al., 2013; Rardin et al., 2013b; Buler et al., 2014). To assess the mammalian succinylome, three independent studies have employed polyclonal succinyl-specific antibodies to enrich for succinylated peptides that were subsequently analyzed by MS/MS approaches (Park et al., 2013; Rardin et al., 2013b; Weinert et al., 2013c). Succinylated proteins were identified with a false-discovery rate (fdr) less than 1%. The focus of each of the studies was (i) mouse liver and HeLa cells (Weinert et al., 2013c), (ii) MLM from wild-type or Sirt5 KO mice, (Rardin et al., 2013b), and (iii) wild-type or Sirt5 KO MEFs (Park et al., 2013). In MEFs, a total of 505 succinylated proteins were identified (Park et al., 2013), whereas in HeLa cells this number reached 738, of which 272 are known mitochondrial proteins. Likewise, in whole mouse liver samples, 750 succinylated proteins were recovered, of which 310 are mitochondrial (Weinert et al., 2013c). Lastly, the analysis of purified MLM yielded 252 succinylated proteins (Rardin et al., 2013b). Collectively, these findings show that lysine succinylation is a frequent modification in mammalian cells whose breadth and cellular localization rival that of acetylation, which prompts the question: do these PTMs target common sites? K-Su/K-Ac overlap appears to be low (~8–10%) in MEFS as well as HeLa cells (12.6%) (Park et al., 2013; Weinert et al., 2013c). By contrast, K-Su/K-Ac overlap was higher in mouse liver (24%) and reached a maximum (38.5%) in purified liver mitochondria (Rardin et al., 2013b; Weinert et al., 2013c). Given that the highest overlap is observed in mitochondria, the variations observed between cell types might reflect differences in their mitochondrial content. Moreover, the extensive overlap inside mitochondria is, again, consistent with the fact that these proteins are in proximity to the source of the acetyl- and succinyl-CoA metabolites (Wagner and Payne, 2013).

### Dynamics and stoichiometry of lysine succinylation

Elucidating the rates with which proteins are succinylated/desuccinylated could lead to better predictions of how this PTM regulates downstream targets. In-depth kinetic studies specifically tailored to assess the dynamics of succinylation in different models and conditions are not yet available. Nevertheless, data from cell-free systems and *in vivo* show that succinylation progresses over the course of hours to days. For example, chemical (i.e., non-enzymatic) succinylation of BSA occurs within 3–6 h (Wagner and Payne, 2013; Weinert et al., 2013c). In cells, pulse-chase experiments with deuterated sodium succinate (D4) determined that succinylation gradually increases within 8–24 h (Xie et al., 2012; Park et al., 2013). In mice, global protein succinylation in liver increases at 48 h of fasting (Park et al., 2013). Moreover, mitochondrial protein succinylation in liver is reduced over the course of several weeks of ethanol exposure (Fritz et al., 2013). While these findings show that succinylation dynamics are slow, they do not rule out the possibility of faster changes under specific conditions. Future studies assessing the kinetics of succinylation/desuccinylation will need to account for the levels and activity of Sirt5 as well as the availability of succinyl-CoA.

Absolute succinylation stoichiometries were assessed in MEFs by a quantitative MS method that uses stable isotope labeling in cells (SILAC) (Park et al., 2013). The majority of quantified proteins had succinylation stoichiometries equal to or less than 10% (Park et al., 2013). Similar stoichiometry trends were obtained for succinylated sites; the site most heavily succinylated (Hspd1 K352) had a stoichiometry close to 80% but the majority of sites had stoichiometries around 15% or below (Park et al., 2013). For reference, the phosphorylation site stoichiometry in dividing HeLa cells is usually above 75% for most substrates (Olsen et al., 2010). Efforts to identify a consensus target sequence, among all succinylation sites, that might imply an enzyme-mediated PTM, have revealed no clear trends (Park et al., 2013; Rardin et al., 2013b; Weinert et al., 2013c). However, examination of sites desuccinylated by Sirt5 reveals a putative target motif in which succinyl lysine is flanked by serines and/or threonines, i.e., (S/T)-K-(S/R) (Rardin et al., 2013b).

### Effects of Sirt5 on cell-wide and mitochondrial succinylation

Succinyl-CoA levels and Sirt5 activity have emerged as the main regulators of protein succinylation and desuccinylation respectively. Analysis of Sirt5 deficient tissues and cells (liver, skeletal muscle, kidney, primary hepatocytes, and MEFs) shows that loss of Sirt5 increases global succinylation, consistent with the proposed role of Sirt5 in repressing succinylation (Peng et al., 2011; Park et al., 2013; Rardin et al., 2013b; Yu et al., 2013). Moreover, Sirt5 deficiency has no effect on global acetylation levels (Peng et al., 2011; Park et al., 2013; Rardin et al., 2013b), nor did Sirt3 deficiency affect global succinylation (Fritz et al., 2013). To identify the *in vivo* substrates of Sirt5, two studies characterized the succinylome in MEFs and MLM with or without Sirt5 (Park et al., 2013; Rardin et al., 2013b). Both studies detect Sirt5 in mitochondria and the cytoplasm, implying that Sirt5 targets both compartments. Furthermore, one study identified about 120 extramitochondrial proteins whose succinylation was regulated by Sirt5 (Park et al., 2013). On the other hand, a separate study did not detect significant succinylation in the cytoplasm of liver extracts or in MEFs by western blot, although it detected prolific succinylation in purified liver mitochondria (Rardin et al., 2013b). One possible explanation for this discrepancy could be the use of different antibodies in the two studies (Park et al., 2013; Rardin et al., 2013b). Nevertheless, despite their differences, these studies provide a comprehensive picture of the Sirt5-regulated succinylome in mammalian cells, a snapshot of which is shown in Table 3 (Park et al., 2013; Rardin et al., 2013b).

**Table 3:**
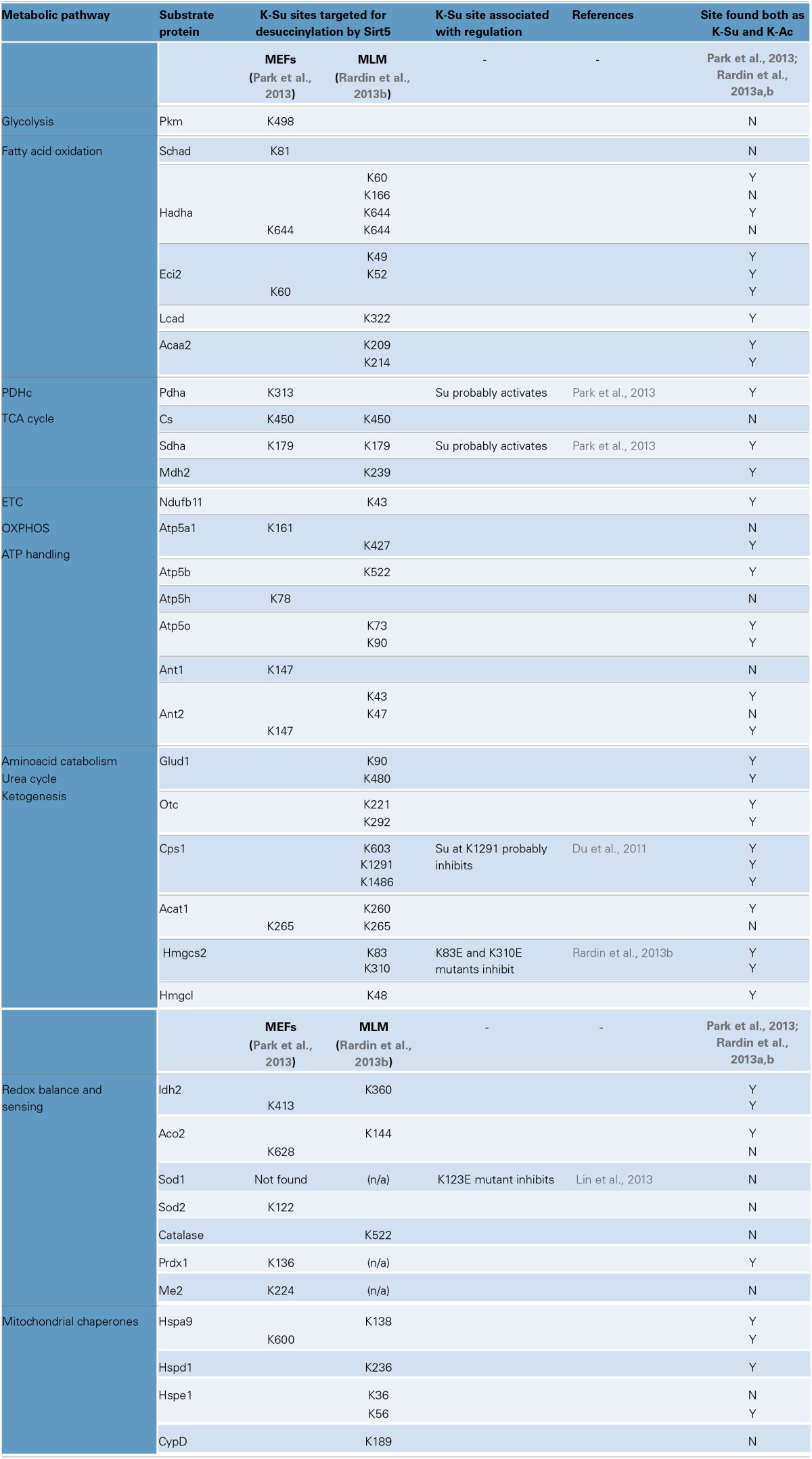
**Protein succinylation, regulation by Sirt5 and functionally important sites**.

*MEFs, mouse embryo fibroblasts; MLM, mouse liver mitochondria; n/a, not applicable (e.g., sod1 is a cytosolic/nuclear protein). All protein names can be referenced to Uniprot for more details. K-to-E are used as succinyl-mimetic mutants to probe whether the residue's succinylation alters enzymatic activity. The table shows a short selection of sites and Sirt5 substrates. For further information the reader is referred to the indicated studies*.

### Regulation of metabolism by succinylation and Sirt5

Prominent targets of succinylation are the major metabolic processes, including the TCA cycle, the pathways breaking down amino acids and fatty acids, and the protein complexes responsible for energy transfer and ATP synthesis. This tropism toward metabolic pathways should not be surprising however, since the majority of studies performed so far focus on liver, or liver-derived material (Du et al., 2011; Fritz et al., 2013; Park et al., 2013; Rardin et al., 2013b; Weinert et al., 2013c). Because succinylation is so widespread, it is challenging to precisely pinpoint the sites that are key to regulation. To this end, defining the Sirt5-regulated succinylome simplifies this quest (Table 3). However, even for this sub-fraction of the succinylome, we lack the mechanistic insights of how succinylation affects function. From a few select examples, it appears that Sirt5-mediated desuccinylation can either enhance, or repress enzymatic activity. According to one study, Sirt5 desuccinylates subunits of the pyruvate dehydrogenase complex (PDHc) and this reduces PDH's activity (Park et al., 2013). A similar mode of regulation is suggested for respiratory complex II (SDH) (Park et al., 2013). If Sirt5 proves to have inhibitory effects on enzymes, this would distinguish it from Sirt3 (activates enzymes by deacetylation). According to a second study however, Sirt5 has activating effects, as it desuccinylates Hmgcs2 to increase hepatic ketogenesis (Rardin et al., 2013b). Sirt5 is also suggested to desuccinylate and activate enzymes regulating fatty acid oxidation in the liver and skeletal muscle (Rardin et al., 2013b). Therefore, though it may be early to suggest that Sirt5-mediated desuccinylation generally opposes actions of Sirt3-mediated deacetylation, it does suggest that lysines targeted by both PTMs may have important regulatory roles (Table 3).

The current evidence suggests that Sirt5 down-regulates glycolysis and the TCA cycle by de-succinylating and inhibiting PDH and SDH complexes (Park et al., 2013), while in separate studies Sirt5-mediated de-succinylation up-regulates fatty acid oxidation, ketogenesis and the urea cycle by activating Hmgcs2, Cps1 and other hepatic enzymes (Du et al., 2011; Rardin et al., 2013b). In the former study, inhibition by Sirt5 was determined in MEFs and extended in HEK cells (Park et al., 2013) while, in the latter, enzyme activation was shown in mouse liver and skeletal muscle and extended to primary hepatocytes, MEFs and HEK cells (Rardin et al., 2013b). Notwithstanding the differences between experimental models and methods, Sirt5 activity might act to influence mitochondrial substrate selectivity, perhaps shunning pyruvate in favor of fatty acids.

Differences are observed regarding the birth ratios of the deficient animals; one strain yields Sirt5^−/−^ animals at normal Mendelian ratios while a second strain yields Sirt5^−/−^ animals at lower than normal ratios (Lombard et al., 2007; Yu et al., 2013). One strain of Sirt5^−/−^ mice is metabolically impaired, exhibiting reduced ketogenesis and fatty acid oxidation during fasting (Rardin et al., 2013b). A second strain however, appears to be metabolically tolerant exhibiting mild improvements in insulin sensitivity in lean and obese states (Yu et al., 2013). Nevertheless, both strains exhibit elevated blood ammonia levels (hyperammonemia) in the fasted state (Nakagawa et al., 2009; Yu et al., 2013). Although all of the above observations are not mutually exclusive, they highlight potential complexities in the regulation of metabolism by Sirt5.

### Protein succinylation and Sirt5 in the heart

These are early days, indeed, for the study of succinylation in the heart. Very little data are available exploring the magnitude of protein succinylation, its implications in protein function, and its impact on cardiac function. The baseline phenotype of Sirt5^−/−^ animals does not reveal significant cardiac abnormalities and several parameters related to heart function, including blood pressure, heart rate, and endurance exercise tolerance are normal (Yu et al., 2013). Nevertheless, mitochondria isolated from Sirt5^−/−^ hearts exhibit considerable increases in global protein succinylation (Tan et al., 2014). At the cellular level, limited data suggest a cytoprotective role for Sirt5 in cardiac myocytes. According to one study, Sirt5 opposes H_2_O_2_–induced death in H9c2 cells and NRVMs (Liu et al., 2013). Mechanistically, the enzymatic activity of Sirt5 appears to be important for the cytoprotective effect and Sirt5 is demonstrated to physically interact with the anti-apoptotic protein Bcl-xL, but not Bcl-2 (Liu et al., 2013). Another study found that Sirt5 protein is upregulated in the heart during intermittent hypoxia (Zhu et al., 2012), a regimen thought to stimulate cardiac tolerance to stress. Collectively, the findings suggest potential roles of Sirt5 in stress-induced cardioprotection, but the limited number of studies and the scarcity of data on cardiac protein succinylation warrant additional investigations before firm conclusions can be made.

## Lysine modification by other dicarboxylates: malonylation and glutarylation

### Malonylation

Malonyl-CoA is a metabolite important in fatty acid biosynthesis, derived from the carboxylation of acetyl-CoA in the cytoplasm and mitochondria (Figure 2). Lysine malonylation (K-Ma) was identified as a new PTM that induces a mass shift of 86 kDa in the spectra of modified peptides (Du et al., 2011; Peng et al., 2011) and, similar to succinylation, malonylation of lysines adds negative charge to the modified residue (see also Figures 2, 3). Moreover, like K-Su, K-Ma is also a substrate for Sirt5 (Du et al., 2011; Peng et al., 2011). Consistently, Sirt5^−/−^ mice exhibit enhanced protein malonylation compared to wild-type mice (Peng et al., 2011). Protein malonylation is detected in *E.coli*, in numerous mammalian cells and tissues; targets include mitochondrial and extramitochondrial proteins (Du et al., 2011; Peng et al., 2011; Xie et al., 2012). Using immunoenrichment followed by LC-MS/MS, 17 malonylated proteins are identified in HeLa cells (Peng et al., 2011). Another study utilizing a membrane-permeable malonate analog, labeled, and identified 375 proteins as candidates for malonylation in HeLa cells (Bao et al., 2013). Interestingly, only a small fraction of these proteins (9%) were known mitochondrial proteins (Bao et al., 2013). This may indicate that malonylation occurs predominantly on extramitochondrial locations, or perhaps that the method is less suitable for targeting of mitochondria. Isotopic labeling with sodium malonate in HeLa cells indicates that protein malonylation occurs within 24 h (mitochondrial proteins Hspa9 and Hspe1 were identified) (Peng et al., 2011). *In vivo*, reductions in protein malonylation (attributable to sirtuin activity) are evident within 2 h in HeLa cells (Bao et al., 2013). As mentioned previously, the dynamics of succinylation depend on the availability of succinyl-CoA (Weinert et al., 2013c). The case may be similar for malonylation as well, where exposure to sodium malonate presumably increases intracellular malonyl-CoA and potentiates protein malonylation (Peng et al., 2011). Finally, the finding that malonylation is under the negative regulation of Sirt5, cautions that current or future phenotypes identified after loss-/gain-of function approaches of Sirt5 in cells and animals, can be attributable to excess protein malonylation (together with succinylation, discussed above and glutarylation, discussed below). To this point, no direct evidence is available that describes the effect of malonylation on substrate function and its role in mammalian physiology.

### Glutarylation

Protein glutarylation is the latest addition to the growing list of lysine acylations identified (Tan et al., 2014). Glutaryl-CoA is produced in mitochondria as a result of the catabolism of tryptophan and lysine (Borsook et al., 1948a,b; Gholson et al., 1962). Glutaryl-CoA contains a negatively charged carboxyl group and is structurally similar to malonyl-CoA and succinyl-CoA. Compared to malonylation and succinylation (identified by a shift of 86 and 100 da respectively), glutarylation causes a shift of 114 Da to the spectra of peptides bearing a modified lysine (Tan et al., 2014). Kinetic and endpoint assays using numerous substrates indicate that Sirt5 has high enzymatic activity for deglutarylation (Tan et al., 2014). The interaction of Sirt5 with malonyl-, succinyl-, and glutaryl-lysines is favored by ionic interactions between the carboxyl group of the substrate, residues forming the substrate binding pocket of Sirt5, as well as the co-substrate, NAD^+^. Moreover, glutaryl-lysine is probably the largest substrate Sirt5 can accommodate, since adipoyl-lysine (which is longer than glutaryl-lysine by one methylene group) is not cleaved by Sirt5 under *in vitro* conditions (Tan et al., 2014).

Conditions favoring protein hyperglutarylation in cells and tissues include: (i) exposure to high concentrations of sodium glutarate, (ii) diet rich in tryptophan, (iii) deficiency of the enzyme catabolizing glutaryl-CoA (glutaryl-CoA dehydrogenase, Gcdh), (iv) prolonged fasting (i.e., for 48 h) and (v) gene deficiency of Sirt5 (Tan et al., 2014). In Sirt5^−/−^ liver lysate, 191 glutarylated proteins are identified by immunoenrichment and HPLC-MS/MS. The majority of them (148), are known mitochondrial proteins which would be consistent with mitochondria as the site of glutaryl-CoA synthesis and Sirt5 as the primary mitochondrial deglutarylase. Glutarylation accumulates on proteins over the course of several hours (within 6 h in enzyme-free reactions and up to 24 h in cells). By contrast, deglutarylation is completed as early as 30 min in the presence of Sirt5 (Tan et al., 2014).

In sum, significant parallels exist in the dynamics and regulation of glutarylation and succinylation (and to a lesser extent, malonylation) illustrating that the role of Sirt5 is more complex than once thought. Among various tissues, protein glutarylation is also detectable in heart mitochondria and it remains to be determined whether this PTM exerts important roles in cardiac function and dysfunction. A first step in addressing these issues would be to determine the baseline levels of glutaryl-CoA in the heart and test whether these fluctuate in response to physiologic and pathologic stimuli. Serving as a genetic model of glutaryl-CoA elevation, Gcdh^−/−^ mice exhibit underlying protein hyperglutarylation (Tan et al., 2014). These mice were found to have fully functional mitochondrial respiratory complexes and normal cardiac histology (Koeller et al., 2002; Sauer et al., 2005). Reassessing the phenotype of these mice in light of protein hyperglutarylation might help identifying important regulatory functions of this PTM in cardiac function.

## Steps toward characterizing propionylation, butyrylation, and crotonylation

Found in the form of thioesters with coenzyme A, propionyl-, butyryl-, and crotonyl- groups are common intermediates in energy metabolism. The breakdown of odd- and even-chain fatty acyl-CoAs through mitochondrial β-oxidation is major source of propionyl-CoA and butyryl-CoA (Figure 2). Moreover, the unsaturated variant, crotonyl-CoA derives from butyryl-CoA or glutaryl-CoA through processing by dedicated mitochondrial dehydrogenases (Green et al., 1954; Lenich and Goodman, 1986). Similarly to acetyl-CoA, propionyl-, butyryl-, and crotonyl-CoA do not have charged groups, which distinguishes them from the dicarboxylate derivatives malonyl-, succinyl-, and glutaryl-CoA which have a negatively charged group. Acetyl-CoA, propionyl-CoA butyryl-CoA, likewise all share the same Gibbs free energy of thioester bond hydrolysis (−36 kJ/mol) (Thauer et al., 1977), and the extended family of acyl-CoA compounds may well exhibit similar reactivity toward lysine residues. Examples of lysine propionylation, butyrylation, and crotonylation (K-Pr, K-Bu, and K-Cr respectively) are identified in bacteria, yeast and mammalian cells. With a few exceptions, most studies focus on the roles these PTMs have on histone and chromatin regulation. Nevertheless, given the mitochondrial origin of propionyl-, butyryl-, and crotonyl-CoAs, it is anticipated that the repertoire of K-pr, K-bu, and K-cr will expand to include mitochondrial proteins as well. Steps toward that direction have already been made (Fritz et al., 2013; Pougovkina et al., 2014b).

### Propionylation

K-Pr results in a shift of 56 Da on the mass of peptides bearing the modification (for comparison, K-Ac causes a shift of 42 da). In the prokaryote *S.enterica*, K-Pr is identified on the enzyme propionyl-CoA synthetase (PrpE) (Garrity et al., 2007). In mammalian cells, K-Pr is found on nuclear proteins p300, CBP, histones H3 and H4 and p53 (Chen et al., 2007; Cheng et al., 2009; Liu et al., 2009). More recently, a total of 42 propionylated proteins were identified in mouse liver, of which 23 came from the mitochondrial fraction (e.g., Acaa2, Cps1, Hmgcs2, catalase) (Fritz et al., 2013). Propionylation likely influences protein function, but specific examples are currently limited. In the case of *S.enterica*, propionylation is suggested to reduce PrpE's enzymatic activity (Garrity et al., 2007). Moreover, high levels of propionyl-CoA inhibit PDHc, αKGDHc and respiratory complex III, although lysine propionylation was not directly tested (Schwab et al., 2006).

A number of studies support that K-Pr is positively regulated by propionyltransferases. The Gcn5-related prokaryotic acetyltransferases Pat and AcuA propionylate PrpE in the presence of propionyl-CoA *in vitro* (Garrity et al., 2007). Moreover, eukaryotic acetyltransferases p300 and Cbp propionylate histones and p53 *in vitro* and *in vivo* (Chen et al., 2007; Cheng et al., 2009; Liu et al., 2009). *In vitro* evidence also shows Pcaf to operate as a propionyltransferase for histone H3 (Leemhuis et al., 2008), although Pcaf was found to be inefficient for protein propionylation in other studies (Chen et al., 2007; Cheng et al., 2009). Nuclear propionyltransferase targets include histone H3K14, H3K23, H4K12, and p53 K292. By comparison, in mitochondria, there is currently no direct evidence for an equivalent enzymatic activity. As suggested for other PTMs, propionyl-CoA levels could be a dominant force shaping the mitochondrial propionylome. Consistently, incubating liver mitochondrial lysates with increasing concentrations of propionyl-CoA elevates protein propionylation (Pougovkina et al., 2014b). Moreover, protein propionylation increases in liver mitochondria of mice exposed to ethanol for 3 or 6 weeks (Fritz et al., 2013) though whether this elevation is the result of propionyltransferase activation, depropionylase inhibition or changes in mitochondrial propionyl-CoA flux, remains to be determined. Regarding dynamics, enzymatic propionylation *in vitro* is completed within 30–120 min (Chen et al., 2007; Garrity et al., 2007; Leemhuis et al., 2008; Liu et al., 2009), whereas chemical propionylation of mitochondrial lysates progresses over the course of 3 h (Pougovkina et al., 2014b).

Negative regulation of protein propionylation by enzymes is a subject of ongoing research. The prokaryotic sirtuin homolog CobB (from the bacterium *S.enterica*) depropionylates PrpE *in vitro*, in a reaction that requires NAD^+^ and produces O-Pr-ADPR (Garrity et al., 2007) (see Figure 3 for similar reactions). PrpE is also depropionylated by another prokaryotic sirtuin homolog (Sir2, from the bacterium *T.maritima*) and by mammalian sirtuins Sirt2 and Sirt3 (Garrity et al., 2007). Measurable *in vitro* depropionylation of synthetic peptides (H3K14-Pr, H3K23-Pr, H3K9-Pr) was also reported for sirtuins Sirt1 and Sirt2, while Sirt3 does not appear as potent (Smith and Denu, 2007; Liu et al., 2009; Feldman et al., 2013). *In vivo*, Sirt1 opposes p53's propionylation in HEK293 cells (Cheng et al., 2009). In liver mitochondria, global protein propionylation is not significantly affected by Sirt3 deficiency (Fritz et al., 2013). Thus, the mitochondrial depropionylase remains elusive, since neither Sirt4 nor Sirt5 are efficient catalysts *in vitro* thus far (Garrity et al., 2007; Feldman et al., 2013).

Little is known regarding the stoichiometry of K-Pr. It is likely that the sites identified thus far represent those with the highest stoichiometry. For reference, K-Pr stoichiometry at H3K23 is estimated to be around 7% in a leukemia cell line (Liu et al., 2009). Moreover, K-Pr on nuclear proteins occurs rather infrequently compared to K-Ac (Cheng et al., 2009). Regarding co-occurrence of acetylation and propionylation at the same K residue (i.e., K-Ac and K-Pr overlap), some information for nuclear proteins suggests that it exists. Moreover, considerable overlap is identified between K-Ac and K-Pr in liver mitochondria (Fritz et al., 2013). The role of lysine propionylation in human disease is only now beginning to be addressed. Deficiency in propionyl-CoA carboxylase (Pcc) in humans results in propionic aciduria, developmental and metabolic defects. Fibroblasts from these patients have protein hyperpropionylation likely resulting from propionyl-CoA accumulation (Pougovkina et al., 2014b). It remains unknown whether protein hyperpropionylation has a causative, or aggravating role in the progression of the disease. The advent of more comprehensive propionylomes will provide a better understanding of this PTM and shed light on its potential implications to cardiac disease.

### Butyrylation

Larger than propionyl-CoA by one methylene group, butyryl-CoA has similarities to propionyl-CoA in terms of its origin, ability to modify lysines and removal by putative de-butyrylases. The first examples of protein lysine butyrylation came by searching for novel PTMs in histone proteomics data. Following a series of validation steps, mass shifts of 70 da in modified peptides were assigned to lysine butyrylation (K-Bu) in histones H3, H4, and nuclear proteins p53, p300, and CBP (Chen et al., 2007; Cheng et al., 2009). In the case of histone H4, butyrylated lysines (e.g., H4K5, H4K8, H4K12, H4K31) were also found to be propionylated, attesting to a significant overlap between K-Bu and K-Pr (Chen et al., 2007). Among 5 HATs, only CBP and p300 can operate as butyryl-transferases *in vitro* and this activity is extended *in vivo* (Chen et al., 2007; Cheng et al., 2009). Owing to CBP and p300 activity, p53 is butyrylated at two positions, K373 and K382 in lung cancer cells (Cheng et al., 2009). Histone butyrylation is also detected in the mouse brain (e.g., H2AK95 and H3K115) (Tweedie-Cullen et al., 2012). There, the stoichiometry of K-Bu at H3K115 was found to be 31%, implying a critical role of butyrylation at this histone site.

In mitochondria, K-Bu is readily detectable by a butyryl-lysine-specific antibody (Fritz et al., 2013). This antibody can be useful in enriching for butyrylated peptides from mitochondrial proteins and identifying them with mass spectrometry. Sirt3 deficiency does not dramatically alter butyrylation in MLM suggesting that Sirt3 is not a major regulator (Fritz et al., 2013), in apparent agreement with *in vitro* studies showing that Sirt3 has low de-butyrylation activity (Smith and Denu, 2007; Feldman et al., 2013). While it is unclear what sirtuin opposes mitochondrial butyrylation (Sirt4 and Sirt5 have no debutyrylase activity; Feldman et al., 2013), emerging evidence suggests that butyryl-CoA flux is a major determinant of butyrylation rates. Genetic deficiency in butyryl-CoA dehydrogenase substantially increases protein butyrylation in liver and this is attributed to aberrant accumulation of hepatic butyryl-CoA (Pougovkina et al., 2014b). Since butyrylation is upregulated, this genetic model could be useful in deciphering the regulatory enzymes opposing protein hyperbutyrylation and also help identify important substrates undergoing butyrylation in various tissues including liver and heart.

### Crotonylation

The identification crotonylation as a novel lysine PTM (68 Da), stems directly from efforts to increase histone protein coverage by derivtizing peptides with propionic anhydride prior to HPLC-MS/MS (Tan et al., 2011). Comparison of the HPLC-MS/MS spectra and elution properties of synthetic and *in vivo* peptides, validated K-Cr as a naturally occurring PTM. This was further substantiated by identifying K-Cr sites after isotopic labeling with D4-crotonate in cells, and with detection of modified proteins using anti-crotonyl-lysine specific antibody (Tan et al., 2011). Examples of K-Cr sites on histones include H2AK36, H2BK5, H3K23, and H4K12. A separate study identifies K-Cr on brain histones, including H2AK95, H2BK108, H3K122, and H4K91, with stoichiometries ranging from 1–3% (Tweedie-Cullen et al., 2012). In contrast to other lysine acylations, K-Cr is structurally more rigid, as the double bond restricts rotation of the carbon atoms. Whether this rigidity has unique implications in histone function remains unknown. One interesting observation is that histone crotonylation is very frequent in gene enhancers and it prevents chromosome inactivation of differentiating sperm cells (Tan et al., 2011).

While the above study laid the framework, questions still remain about the regulation of K-Cr and its impact on non-histone and perhaps mitochondrial proteins. The dynamics of K-Cr are likely governed by crotonyl-CoA levels. Altering the flux of crotonyl-CoA (a metabolic intermediate in fatty acid and amino acid catabolism) could directly affect global mitochondrial protein crotonylation. Studies are also designed to identify enzymatic activities promoting or opposing crotonylation. In contrast to what has been shown for propionylation and butyrylation, the HATs p300 and CBP do not promote crotonylation (Tan et al., 2011). Furthermore, none of the 11 histone deacetylases (HDACs) can decrotonylate efficiently (Tan et al., 2011). A screen of the sirtuins for decrotonylation activity using a synthetic peptide (H4K12-Cr) found Sirt1 to be somewhat active, while Sirt2, 3, 5 and 6 had negligible activity (Madsen and Olsen, 2012). A separate study using a different substrate (H3K9-Cr) found measurable decrotonylase activities in Sirt1 and Sirt2 but not in Sirt3-Sirt6 (Feldman et al., 2013). In aggregate, sirtuins Sirt3-Sirt5 are unlikely to be decrotonylases, so a dedicated mitochondrial decrotonylase remains to be discovered. An alternative possibility is that the crotonyl moiety is modified *in situ* to become the substrate of one of the mitochondrial sirtuins (e.g., conversion of K-Cr to K-Ac through a series of enzymatic steps and final deacetylation by Sirt3).

The importance of K-Cr to mitochondrial protein activity has yet to be established. A genetic model of protein hypercrotonylation would be valuable in this regard, just as Gcdh and Bcdh-null models have provided insights into the roles hyperglutarylation and hyperbutyrylation respectively (Pougovkina et al., 2014b; Tan et al., 2014). Protein hypercrotonylation might be possible by inactivating crotonase, a multisubunit enzyme converting crotonyl-CoA to 3-hydroxybutyryl-CoA (Engel et al., 1996). One limitation could be that the levels of enoyl-CoA intermediates other than crotonyl-CoA might be affected, since crotonase has broad specificity (Waterson and Hill, 1972; Schulz, 1974; Fong and Schulz, 1977). More investigation is warranted for this newly discovered PTM and its potential implications in mitochondrial protein regulation in heart and other tissues.

## Conclusions

Once exclusively the purview of histone research, the study of lysine PTMs has undergone a profound maturation. Mitochondria are epicenters for an evolving class of lysine PTMs including succinylation, malonylation, and glutarylation. The result has been a data deluge. And so begins the daunting task of piecing it all together and sorting the regulatory PTMs from the epiphenomena of metabolic stress (Beltrao et al., 2012). And with these discoveries comes the realization that newer tools may be required to assess the causal relationship between PTM and protein function. For instance, until now, mutation of K to Q has served as an acceptable mimic of the charge neutralization of acetylation, while K to E mutation has served to mimic the negative charge of succinylation. Are these mutations equally suitable for the study of propionylation, malonylation, and glutarylation, whose size differ? Promisingly, Neumann et al. have developed a method to introduce site-specific acetylated lysine residues in recombinant proteins expressed in *E.coli* (Neumann et al., 2008). If the method can be honed to incorporate other modified lysines, it will be possible to test their effect on function and the extent to which K to Q/E mutation may serve as a valid proxy in mammalian cell culture.

The new lysine modifications join the pantheon of metabogenic PTMs, including O-GlcNacylation and palmitoylation among others. And while great strides have been made to understand how specific nutrients act as ligands to activate transcriptional programs to modulate metabolism, it is also clear now that metabolism, itself, leaves its mark on the proteome in a manner that ultimately affects the activity of enzymes. This increased complexity and nuanced regulation may afford new opportunities for therapeutic intervention in disease and the mitochondrial sirtuins promise to be prime targets. Already, emerging evidence shows that increasing the availability of NAD^+^ (e.g., through dietary supplementation of its biosynthetic intermediates) can boost sirtuin activity and improve mitochondrial function, conferring salutary effects in diabetes and aging (Yoshino et al., 2011; Canto et al., 2012; Mouchiroud et al., 2013). As we delve deeper into the role of newly-found lysine modifications in heart disease, it may also be desirable to develop chemical screens for Sirt3 and Sirt5-specific modulators.

## Sources of funding

D. Brian Foster is supported by NIH R21HL108052, an American Heart Association National Scientist Development Grant, 12SDG12060056, and funding from the Zegar Family Foundation. Brian O'Rourke acknowledges funding from HHSN268201000032C.

### Conflict of interest statement

The authors declare that the research was conducted in the absence of any commercial or financial relationships that could be construed as a potential conflict of interest.

## References

[B1] Ahn B. H. Kim H. S. Song S. Lee I. H. Liu J. Vassilopoulos A. (2008). A role for the mitochondrial deacetylase Sirt3 in regulating energy homeostasis. Proc. Natl. Acad. Sci. U.S.A. 105, 14447–14452 10.1073/pnas.0803790105 18794531 PMC2567183

[B2] Ahuja N. Schwer B. Carobbio S. Waltregny D. North B. J. Castronovo V. (2007). Regulation of insulin secretion by SIRT4, a mitochondrial ADP-ribosyltransferase. J. Biol. Chem. 282, 33583–33592 10.1074/jbc.M705488200 17715127

[B3] Allfrey V. Faulkner R. Mirsky A. E. (1964). Acetylation and methylation of histones and their possible role in the regulation of RNA synthesis. Proc. Natl. Acad. Sci. U.S.A. 51, 786–794 10.1073/pnas.51.5.786 14172992 PMC300163

[B4] Baeza J. Dowell J. A. Smallegan M. J. Fan J. Amador-Noguez D. Khan Z. (2014). Stoichiometry of site-specific lysine acetylation in an entire proteome. J. Biol. Chem. 289, 21326–21338 10.1074/jbc.M114.581843 24917678 PMC4118097

[B5] Bao J. Lu Z. Joseph J. J. Carabenciov D. Dimond C. C. Pang L. (2010). Characterization of the murine SIRT3 mitochondrial localization sequence and comparison of mitochondrial enrichment and deacetylase activity of long and short SIRT3 isoforms. J. Cell. Biochem. 110, 238–247 10.1002/jcb.22531 20235147 PMC2858784

[B6] Bao X. Zhao Q. Yang T. Fung Y. M. Li X. D. (2013). A chemical probe for lysine malonylation. Angewandte Chemie 52, 4883–4886 10.1002/anie.201300252 23533089

[B7] Beltrao P. Albanese V. Kenner L. R. Swaney D. L. Burlingame A. Villen J. (2012). Systematic functional prioritization of protein posttranslational modifications. Cell 150, 413–425 10.1016/j.cell.2012.05.036 22817900 PMC3404735

[B8] Bharathi S. S. Zhang Y. Mohsen A. W. Uppala R. Balasubramani M. Schreiber E. (2013). Sirtuin 3 (SIRT3) protein regulates long-chain acyl-CoA dehydrogenase by deacetylating conserved lysines near the active site. J. Biol. Chem. 288, 33837–33847 10.1074/jbc.M113.510354 24121500 PMC3837126

[B9] Borsook H. Deasy C. L. Haagen-Smit A. J. Keighley G. Lowy P. H. (1948a). The degradation of l-lysine in guinea pig liver homogenate; formation of alpha-aminoadipic acid. J. Biol. Chem. 176, 1383–1393 18098588

[B10] Borsook H. Deasy C. L. Haagen-Smit A. J. Keighley G. Lowy P. H. (1948b). The degradation of alpha-aminoadipic acid in guinea pig liver homogenate. J. Biol. Chem. 176, 1395–1400 18098589

[B11] Buler M. Aatsinki S. M. Izzi V. Uusimaa J. Hakkola J. (2014). SIRT5 is under the control of PGC-1alpha and AMPK and is involved in regulation of mitochondrial energy metabolism. FASEB J. 28, 3225–3237 10.1096/fj.13-245241 24687991

[B12] Canto C. Houtkooper R. H. Pirinen E. Youn D. Y. Oosterveer M. H. Cen Y. (2012). The NAD(+) precursor nicotinamide riboside enhances oxidative metabolism and protects against high-fat diet-induced obesity. Cell Metab. 15, 838–847 10.1016/j.cmet.2012.04.022 22682224 PMC3616313

[B13] Chen C. J. Fu Y. C. Yu W. Wang W. (2013). SIRT3 protects cardiomyocytes from oxidative stress-mediated cell death by activating NF-kappaB. Biochem. Biophys. Res. Commun. 430, 798–803 10.1016/j.bbrc.2012.11.066 23201401

[B14] Chen Y. Sprung R. Tang Y. Ball H. Sangras B. Kim S. C. (2007). Lysine propionylation and butyrylation are novel post-translational modifications in histones. Mol. Cell. Proteomics 6, 812–819 10.1074/mcp.M700021-MCP200 17267393 PMC2911958

[B15] Chen Y. Zhang J. Lin Y. Lei Q. Guan K. L. Zhao S. (2011). Tumour suppressor SIRT3 deacetylates and activates manganese superoxide dismutase to scavenge ROS. EMBO Rep. 12, 534–541 10.1038/embor.2011.65 21566644 PMC3128277

[B16] Cheng Z. Tang Y. Chen Y. Kim S. Liu H. Li S. S. (2009). Molecular characterization of propionyllysines in non-histone proteins. Mol. Cell. Proteomics 8, 45–52 10.1074/mcp.M800224-MCP200 18753126 PMC2621001

[B17] Choudhary C. Kumar C. Gnad F. Nielsen M. L. Rehman M. Walther T. C. (2009). Lysine acetylation targets protein complexes and co-regulates major cellular functions. Science 325, 834–840 10.1126/science.1175371 19608861

[B18] Colak G. Xie Z. Zhu A. Y. Dai L. Lu Z. Zhang Y. (2013). Identification of lysine succinylation substrates and the succinylation regulatory enzyme CobB in *Escherichia coli*. Mol. Cell. Proteomics 12, 3509–3520 10.1074/mcp.M113.031567 24176774 PMC3861704

[B19] Csibi A. Fendt S. M. Li C. Poulogiannis G. Choo A. Y. Chapski D. J. (2013). The mTORC1 pathway stimulates glutamine metabolism and cell proliferation by repressing SIRT4. Cell 153, 840–854 10.1016/j.cell.2013.04.023 23663782 PMC3684628

[B20] Du J. Jiang H. Lin H. (2009). Investigating the ADP-ribosyltransferase activity of sirtuins with NAD analogues and 32P-NAD. Biochemistry 48, 2878–2890 10.1021/bi802093g 19220062

[B21] Du J. Zhou Y. Su X. Yu J. J. Khan S. Jiang H. (2011). Sirt5 is a NAD-dependent protein lysine demalonylase and desuccinylase. Science 334, 806–809 10.1126/science.1207861 22076378 PMC3217313

[B22] Elrod J. W. Wong R. Mishra S. Vagnozzi R. J. Sakthievel B. Goonasekera S. A. (2010). Cyclophilin D controls mitochondrial pore-dependent Ca(2+) exchange, metabolic flexibility, and propensity for heart failure in mice. J. Clin. Invest. 120, 3680–3687 10.1172/JCI43171 20890047 PMC2947235

[B23] Engel C. K. Mathieu M. Zeelen J. P. Hiltunen J. K. Wierenga R. K. (1996). Crystal structure of enoyl-coenzyme A (CoA) hydratase at 2.5 angstroms resolution: a spiral fold defines the CoA-binding pocket. EMBO J. 15, 5135–5145 8895557 PMC452256

[B24] Feldman J. L. Baeza J. Denu J. M. (2013). Activation of the protein deacetylase SIRT6 by long-chain fatty acids and widespread deacylation by mammalian sirtuins. J. Biol. Chem. 288, 31350–31356 10.1074/jbc.C113.511261 24052263 PMC3829447

[B25] Fernandez-Marcos P. J. Jeninga E. H. Canto C. Harach T. de Boer V. C. Andreux P. (2012). Muscle or liver-specific Sirt3 deficiency induces hyperacetylation of mitochondrial proteins without affecting global metabolic homeostasis. Sci. Rep. 2:425 10.1038/srep00425 22645641 PMC3361023

[B26] Fischer F. Gertz M. Suenkel B. Lakshminarasimhan M. Schutkowski M. Steegborn C. (2012). Sirt5 deacylation activities show differential sensitivities to nicotinamide inhibition. PLoS ONE 7:e45098 10.1371/journal.pone.0045098 23028781 PMC3446968

[B27] Fong J. C. Schulz H. (1977). Purification and properties of pig heart crotonase and the presence of short chain and long chain enoyl coenzyme A hydratases in pig and guinea pig tissues. J. Biol. Chem. 252, 542–547 833142

[B28] Foster D. B. Liu T. Rucker J. O'Meally R. N. Devine L. R. Cole R. N. (2013). The cardiac acetyl-lysine proteome. PLoS ONE 8:e67513 10.1371/journal.pone.0067513 23844019 PMC3699649

[B29] Foster D. B. Van Eyk J. E. Marban E. O'Rourke B. (2009). Redox signaling and protein phosphorylation in mitochondria: progress and prospects. J. Bioenerg. Biomembr. 41, 159–168 10.1007/s10863-009-9217-7 19440831 PMC2921908

[B30] Fritz K. S. Green M. F. Petersen D. R. Hirschey M. D. (2013). Ethanol metabolism modifies hepatic protein acylation in mice. PLoS ONE 8:e75868 10.1371/journal.pone.0075868 24073283 PMC3779192

[B31] Garrity J. Gardner J. G. Hawse W. Wolberger C. Escalante-Semerena J. C. (2007). N-lysine propionylation controls the activity of propionyl-CoA synthetase. J. Biol. Chem. 282, 30239–30245 10.1074/jbc.M704409200 17684016

[B32] Gershey E. L. Vidali G. Allfrey V. G. (1968). Chemical studies of histone acetylation. The occurrence of epsilon-N-acetyllysine in the f2a1 histone. J. Biol. Chem. 243, 5018–5022 5679978

[B33] Gholson R. K. Nishizuka Y. Ichiyama A. Kawai H. Nakamura S. Hayaishi O. (1962). New intermediates in the catabolism of tryptophan in mammalian liver. J. Biol. Chem. 237, 2043–2045 13898215

[B34] Green D. E. Mii S. Mahler H. R. Bock R. M. (1954). Studies on the fatty acid oxidizing system of animal tissues. III. Butyryl coenzyme A dehydrogenase. J. Biol. Chem. 206, 1–12 13130521

[B35] Grillon J. M. Johnson K. R. Kotlo K. Danziger R. S. (2012). Non-histone lysine acetylated proteins in heart failure. Biochim. Biophys. Acta 1822, 607–614 10.1016/j.bbadis.2011.11.016 22155497 PMC3684243

[B36] Hafner A. V. Dai J. Gomes A. P. Xiao C. Y. Palmeira C. M. Rosenzweig A. (2010). Regulation of the mPTP by SIRT3-mediated deacetylation of CypD at lysine 166 suppresses age-related cardiac hypertrophy. Aging 2, 914–923 21212461 10.18632/aging.100252 PMC3034180

[B37] Haigis M. C. Guarente L. P. (2006). Mammalian sirtuins–emerging roles in physiology, aging, and calorie restriction. Genes Dev. 20, 2913–2921 10.1101/gad.1467506 17079682

[B38] Haigis M. C. Mostoslavsky R. Haigis K. M. Fahie K. Christodoulou D. C. Murphy A. J. (2006). SIRT4 inhibits glutamate dehydrogenase and opposes the effects of calorie restriction in pancreatic beta cells. Cell 126, 941–954 10.1016/j.cell.2006.06.057 16959573

[B39] Hallows W. C. Lee S. Denu J. M. (2006). Sirtuins deacetylate and activate mammalian acetyl-CoA synthetases. Proc. Natl. Acad. Sci. U.S.A. 103, 10230–10235 10.1073/pnas.0604392103 16790548 PMC1480596

[B40] Hallows W. C. Yu W. Smith B. C. Devries M. K. Ellinger J. J. Someya S. (2011). Sirt3 promotes the urea cycle and fatty acid oxidation during dietary restriction. Mol. Cell 41, 139–149 10.1016/j.molcel.2011.01.002 21255725 PMC3101115

[B41] Hebert A. S. Dittenhafer-Reed K. E. Yu W. Bailey D. J. Selen E. S. Boersma M. D. (2013). Calorie restriction and SIRT3 trigger global reprogramming of the mitochondrial protein acetylome. Mol. Cell 49, 186–199 10.1016/j.molcel.2012.10.024 23201123 PMC3704155

[B42] Henriksen P. Wagner S. A. Weinert B. T. Sharma S. Bacinskaja G. Rehman M. (2012). Proteome-wide analysis of lysine acetylation suggests its broad regulatory scope in *Saccharomyces cerevisiae*. Mol. Cell. Proteomics 11, 1510–1522 10.1074/mcp.M112.017251 22865919 PMC3494197

[B43] Hirschey M. D. Shimazu T. Goetzman E. Jing E. Schwer B. Lombard D. B. (2010). SIRT3 regulates mitochondrial fatty-acid oxidation by reversible enzyme deacetylation. Nature 464, 121–125 10.1038/nature08778 20203611 PMC2841477

[B44] Hirschey M. D. Shimazu T. Jing E. Grueter C. A. Collins A. M. Aouizerat B. (2011). SIRT3 deficiency and mitochondrial protein hyperacetylation accelerate the development of the metabolic syndrome. Mol. Cell 44, 177–190 10.1016/j.molcel.2011.07.019 21856199 PMC3563434

[B45] Ho L. Titus A. S. Banerjee K. K. George S. Lin W. Deota S. (2013). SIRT4 regulates ATP homeostasis and mediates a retrograde signaling via AMPK. Aging 5, 835–849 24296486 10.18632/aging.100616 PMC3868726

[B46] Jeong S. M. Xiao C. Finley L. W. Lahusen T. Souza A. L. Pierce K. (2013). SIRT4 has tumor-suppressive activity and regulates the cellular metabolic response to DNA damage by inhibiting mitochondrial glutamine metabolism. Cancer Cell 23, 450–463 10.1016/j.ccr.2013.02.024 23562301 PMC3650305

[B47] Jiang W. Wang S. Xiao M. Lin Y. Zhou L. Lei Q. (2011). Acetylation regulates gluconeogenesis by promoting PEPCK1 degradation via recruiting the UBR5 ubiquitin ligase. Mol. Cell 43, 33–44 10.1016/j.molcel.2011.04.028 21726808 PMC3962309

[B48] Karamanlidis G. Lee C. F. Garcia-Menendez L. Kolwicz S. C. Jr. Suthammarak W. Gong G. (2013). Mitochondrial complex I deficiency increases protein acetylation and accelerates heart failure. Cell Metab. 18, 239–250 10.1016/j.cmet.2013.07.002 23931755 PMC3779647

[B49] Kim D. Yu B. J. Kim J. A. Lee Y. J. Choi S. G. Kang S. (2013). The acetylproteome of Gram-positive model bacterium *Bacillus subtilis*. Proteomics 13, 1726–1736 10.1002/pmic.201200001 23468065

[B50] Kim S. C. Sprung R. Chen Y. Xu Y. Ball H. Pei J. (2006). Substrate and functional diversity of lysine acetylation revealed by a proteomics survey. Mol. Cell 23, 607–618 10.1016/j.molcel.2006.06.026 16916647

[B51] Koeller D. M. Woontner M. Crnic L. S. Kleinschmidt-DeMasters B. Stephens J. Hunt E. L. (2002). Biochemical, pathologic and behavioral analysis of a mouse model of glutaric acidemia type I. Hum. Mol. Genet. 11, 347–357 10.1093/hmg/11.4.347 11854167

[B52] Laurent G. de Boer V. C. Finley L. W. Sweeney M. Lu H. Schug T. T. (2013b). SIRT4 represses peroxisome proliferator-activated receptor alpha activity to suppress hepatic fat oxidation. Mol. Cell. Biol. 33, 4552–4561 10.1128/MCB.00087-13 24043310 PMC3838178

[B53] Laurent G. German N. J. Saha A. K. de Boer V. C. Davies M. Koves T. R. (2013a). SIRT4 coordinates the balance between lipid synthesis and catabolism by repressing malonyl CoA decarboxylase. Mol. Cell 50, 686–698 10.1016/j.molcel.2013.05.012 23746352 PMC3721068

[B54] Lebovitz R. M. Zhang H. Vogel H. Cartwright J. Jr. Dionne L. Lu N. (1996). Neurodegeneration, myocardial injury, and perinatal death in mitochondrial superoxide dismutase-deficient mice. Proc. Natl. Acad. Sci. U.S.A. 93, 9782–9787 10.1073/pnas.93.18.9782 8790408 PMC38506

[B55] Leemhuis H. Packman L. C. Nightingale K. P. Hollfelder F. (2008). The human histone acetyltransferase P/CAF is a promiscuous histone propionyltransferase. Chembiochem 9, 499–503 10.1002/cbic.200700556 18247445

[B56] Lenich A. C. Goodman S. I. (1986). The purification and characterization of glutaryl-coenzyme A dehydrogenase from porcine and human liver. J. Biol. Chem. 261, 4090–4096 3081514

[B57] Lima B. P. Thanh Huyen T. T. Basell K. Becher D. Antelmann H. Wolfe A. J. (2012). Inhibition of acetyl phosphate-dependent transcription by an acetylatable lysine on RNA polymerase. J. Biol. Chem. 287, 32147–32160 10.1074/jbc.M112.365502 22829598 PMC3442545

[B58] Lin Z. F. Xu H. B. Wang J. Y. Lin Q. Ruan Z. Liu F. B. (2013). SIRT5 desuccinylates and activates SOD1 to eliminate ROS. Biochem. Biophys. Res. Commun. 441, 191–195 10.1016/j.bbrc.2013.10.033 24140062

[B59] Liu B. Che W. Zheng C. Liu W. Wen J. Fu H. (2013). SIRT5: a safeguard against oxidative stress-induced apoptosis in cardiomyocytes. Cell. Physiol. Biochem. 32, 1050–1059 10.1159/000354505 24192575

[B60] Liu B. Lin Y. Darwanto A. Song X. Xu G. Zhang K. (2009). Identification and characterization of propionylation at histone H3 lysine 23 in mammalian cells. J. Biol. Chem. 284, 32288–32295 10.1074/jbc.M109.045856 19801601 PMC2781642

[B61] Lombard D. B. Alt F. W. Cheng H. L. Bunkenborg J. Streeper R. S. Mostoslavsky R. (2007). Mammalian Sir2 homolog SIRT3 regulates global mitochondrial lysine acetylation. Mol. Cell. Biol. 27, 8807–8814 10.1128/MCB.01636-07 17923681 PMC2169418

[B62] Lundby A. Lage K. Weinert B. T. Bekker-Jensen D. B. Secher A. Skovgaard T. (2012). Proteomic analysis of lysine acetylation sites in rat tissues reveals organ specificity and subcellular patterns. Cell Rep. 2, 419–431 10.1016/j.celrep.2012.07.006 22902405 PMC4103158

[B63] Madsen A. S. Olsen C. A. (2012). Substrates for efficient fluorometric screening employing the NAD-dependent sirtuin 5 lysine deacylase (KDAC) enzyme. J. Med. Chem. 55, 5582–5590 10.1021/jm300526r 22583019

[B64] Mailloux R. J. Jin X. Willmore W. G. (2014). Redox regulation of mitochondrial function with emphasis on cysteine oxidation reactions. Redox Biol. 2, 123–139 10.1016/j.redox.2013.12.011 24455476 PMC3895620

[B65] Michishita E. Park J. Y. Burneskis J. M. Barrett J. C. Horikawa I. (2005). Evolutionarily conserved and nonconserved cellular localizations and functions of human SIRT proteins. Mol. Biol. Cell 16, 4623–4635 10.1091/mbc.E05-01-0033 16079181 PMC1237069

[B66] Mouchiroud L. Houtkooper R. H. Moullan N. Katsyuba E. Ryu D. Canto C. (2013). The NAD(+)/sirtuin pathway modulates longevity through activation of mitochondrial UPR and FOXO signaling. Cell 154, 430–441 10.1016/j.cell.2013.06.016 23870130 PMC3753670

[B67] Nadtochiy S. M. Redman E. Rahman I. Brookes P. S. (2011). Lysine deacetylation in ischaemic preconditioning: the role of SIRT1. Cardiovasc. Res. 89, 643–649 10.1093/cvr/cvq287 20823277 PMC3028968

[B68] Nakagawa T. Lomb D. J. Haigis M. C. Guarente L. (2009). SIRT5 Deacetylates carbamoyl phosphate synthetase 1 and regulates the urea cycle. Cell 137, 560–570 10.1016/j.cell.2009.02.026 19410549 PMC2698666

[B69] Neumann H. Peak-Chew S. Y. Chin J. W. (2008). Genetically encoding N(epsilon)-acetyllysine in recombinant proteins. Nat. Chem. Biol. 4, 232–234 10.1038/nchembio.73 18278036

[B70] Newman J. C. He W. Verdin E. (2012). Mitochondrial protein acylation and intermediary metabolism: regulation by sirtuins and implications for metabolic disease. J. Biol. Chem. 287, 42436–42443 10.1074/jbc.R112.404863 23086951 PMC3522244

[B71] Nguyen T. T. Wong R. Menazza S. Sun J. Chen Y. Wang G. (2013). Cyclophilin D modulates mitochondrial acetylome. Circ. Res. 113, 1308–1319 10.1161/CIRCRESAHA.113.301867 24062335 PMC4180423

[B72] Olsen J. V. Vermeulen M. Santamaria A. Kumar C. Miller M. L. Jensen L. J. (2010). Quantitative phosphoproteomics reveals widespread full phosphorylation site occupancy during mitosis. Sci. Signal. 3, ra3 10.1126/scisignal.2000475 20068231

[B73] Onyango P. Celic I. McCaffery J. M. Boeke J. D. Feinberg A. P. (2002). SIRT3, a human SIR2 homologue, is an NAD-dependent deacetylase localized to mitochondria. Proc. Natl. Acad. Sci. U.S.A. 99, 13653–13658 10.1073/pnas.222538099 12374852 PMC129731

[B74] O'Rourke B. Van Eyk J. E. Foster D. B. (2011). Mitochondrial protein phosphorylation as a regulatory modality: implications for mitochondrial dysfunction in heart failure. Congest. Heart Fail. 17, 269–282 10.1111/j.1751-7133.2011.00266.x 22103918 PMC4067253

[B75] Park J. Chen Y. Tishkoff D. X. Peng C. Tan M. Dai L. (2013). SIRT5-mediated lysine desuccinylation impacts diverse metabolic pathways. Mol. Cell 50, 919–930 10.1016/j.molcel.2013.06.001 23806337 PMC3769971

[B76] Peng C. Lu Z. Xie Z. Cheng Z. Chen Y. Tan M. (2011). The first identification of lysine malonylation substrates and its regulatory enzyme. Mol. Cell. Proteomics 10:M111.012658 10.1074/mcp.M111.012658 21908771 PMC3237090

[B77] Porter G. Urciuoli W. R. Brookes P. S. Nadtochiy S. M. (2014). SIRT3 deficiency exacerbates ischemia-reperfusion injury: implication for aged hearts. Am. J. Physiol. Heart Circ. Physiol. 306, H1602–H1609 10.1152/ajpheart.00027.2014 24748594 PMC4059981

[B78] Pougovkina O. Te Brinke H. Ofman R. van Cruchten A. G. Kulik W. Wanders R. J. (2014a). Mitochondrial protein acetylation is driven by acetyl-CoA from fatty acid oxidation. Hum. Mol. Genet. 23, 3513–3522 10.1093/hmg/ddu059 24516071

[B79] Pougovkina O. Te Brinke H. Wanders R. J. Houten S. M. de Boer V. C. (2014b). Aberrant protein acylation is a common observation in inborn errors of acyl-CoA metabolism. J. Inherit. Metab. Dis. [Epub ahead of print]. 10.1007/s10545-014-9684-9 24531926

[B80] Puccio H. Simon D. Cossee M. Criqui-Filipe P. Tiziano F. Melki J. (2001). Mouse models for Friedreich ataxia exhibit cardiomyopathy, sensory nerve defect and Fe-S enzyme deficiency followed by intramitochondrial iron deposits. Nat. Genet. 27, 181–186 10.1038/84818 11175786

[B81] Qiu X. Brown K. Hirschey M. D. Verdin E. Chen D. (2010). Calorie restriction reduces oxidative stress by SIRT3-mediated SOD2 activation. Cell Metab. 12, 662–667 10.1016/j.cmet.2010.11.015 21109198

[B82] Rardin M. J. He W. Nishida Y. Newman J. C. Carrico C. Danielson S. R. (2013b). SIRT5 regulates the mitochondrial lysine succinylome and metabolic networks. Cell Metab. 18, 920–933 10.1016/j.cmet.2013.11.013 24315375 PMC4105152

[B83] Rardin M. J. Newman J. C. Held J. M. Cusack M. P. Sorensen D. J. Li B. (2013a). Label-free quantitative proteomics of the lysine acetylome in mitochondria identifies substrates of SIRT3 in metabolic pathways. Proc. Natl. Acad. Sci. U.S.A. 110, 6601–6606 10.1073/pnas.1302961110 23576753 PMC3631688

[B84] Rauh D. Fischer F. Gertz M. Lakshminarasimhan M. Bergbrede T. Aladini F. (2013). An acetylome peptide microarray reveals specificities and deacetylation substrates for all human sirtuin isoforms. Nat. Commun. 4, 2327 10.1038/ncomms3327 23995836

[B85] Rosca M. G. Lemieux H. Hoppel C. L. (2009). Mitochondria in the elderly: Is acetylcarnitine a rejuvenator? Adv. Drug Deliv. Rev. 61, 1332–1342 10.1016/j.addr.2009.06.009 19720100 PMC4120470

[B86] Samant S. A. Zhang H. J. Hong Z. Pillai V. B. Sundaresan N. R. Wolfgeher D. (2013). SIRT3 deacetylates and activates OPA1 to regulate mitochondrial dynamics during stress. Mol. Cell. Biol. 34, 807–819 10.1128/MCB.01483-13 24344202 PMC4023816

[B87] Sauer S. W. Okun J. G. Schwab M. A. Crnic L. R. Hoffmann G. F. Goodman S. I. (2005). Bioenergetics in glutaryl-coenzyme A dehydrogenase deficiency: a role for glutaryl-coenzyme A. J. Biol. Chem. 280, 21830–21836 10.1074/jbc.M502845200 15840571

[B88] Schulz H. (1974). Long chain enoyl coenzyme A hydratase from pig heart. J. Biol. Chem. 249, 2704–2709 4828315

[B89] Schwab M. A. Sauer S. W. Okun J. G. Nijtmans L. G. Rodenburg R. J. van den Heuvel L. P. (2006). Secondary mitochondrial dysfunction in propionic aciduria: a pathogenic role for endogenous mitochondrial toxins. Biochem. J. 398, 107–112 10.1042/BJ20060221 16686602 PMC1525008

[B90] Schwer B. Bunkenborg J. Verdin R. O. Andersen J. S. Verdin E. (2006). Reversible lysine acetylation controls the activity of the mitochondrial enzyme acetyl-CoA synthetase 2. Proc. Natl. Acad. Sci. U.S.A. 103, 10224–10229 10.1073/pnas.0603968103 16788062 PMC1502439

[B91] Schwer B. Eckersdorff M. Li Y. Silva J. C. Fermin D. Kurtev M. V. (2009). Calorie restriction alters mitochondrial protein acetylation. Aging Cell 8, 604–606 10.1111/j.1474-9726.2009.00503.x 19594485 PMC2752488

[B92] Schwer B. North B. J. Frye R. A. Ott M. Verdin E. (2002). The human silent information regulator (Sir)2 homologue hSIRT3 is a mitochondrial nicotinamide adenine dinucleotide-dependent deacetylase. J. Cell Biol. 158, 647–657 10.1083/jcb.200205057 12186850 PMC2174009

[B93] Scott I. Webster B. R. Li J. H. Sack M. N. (2012). Identification of a molecular component of the mitochondrial acetyltransferase programme: a novel role for GCN5L1. Biochem. J. 443, 655–661 10.1042/BJ20120118 22309213 PMC7461726

[B94] Shi T. Wang F. Stieren E. Tong Q. (2005). SIRT3, a mitochondrial sirtuin deacetylase, regulates mitochondrial function and thermogenesis in brown adipocytes. J. Biol. Chem. 280, 13560–13567 10.1074/jbc.M414670200 15653680

[B95] Shimazu T. Hirschey M. D. Hua L. Dittenhafer-Reed K. E. Schwer B. Lombard D. B. (2010). SIRT3 deacetylates mitochondrial 3-hydroxy-3-methylglutaryl CoA synthase 2 and regulates ketone body production. Cell Metab. 12, 654–661 10.1016/j.cmet.2010.11.003 21109197 PMC3310379

[B96] Shinmura K. Tamaki K. Sano M. Nakashima-Kamimura N. Wolf A. M. Amo T. (2011). Caloric restriction primes mitochondria for ischemic stress by deacetylating specific mitochondrial proteins of the electron transport chain. Circ. Res. 109, 396–406 10.1161/CIRCRESAHA.111.243097 21700931

[B97] Shulga N. Wilson-Smith R. Pastorino J. G. (2010). Sirtuin-3 deacetylation of cyclophilin D induces dissociation of hexokinase II from the mitochondria. J. Cell Sci. 123, 894–902 10.1242/jcs.061846 20159966 PMC3189253

[B98] Smith B. C. Denu J. M. (2007). Acetyl-lysine analog peptides as mechanistic probes of protein deacetylases. J. Biol. Chem. 282, 37256–37265 10.1074/jbc.M707878200 17951578

[B99] Sol E. M. Wagner S. A. Weinert B. T. Kumar A. Kim H. S. Deng C. X. (2012). Proteomic investigations of lysine acetylation identify diverse substrates of mitochondrial deacetylase sirt3. PLoS ONE 7:e50545 10.1371/journal.pone.0050545 23236377 PMC3517600

[B100] Someya S. Yu W. Hallows W. C. Xu J. Vann J. M. Leeuwenburgh C. (2010). Sirt3 mediates reduction of oxidative damage and prevention of age-related hearing loss under caloric restriction. Cell 143, 802–812 10.1016/j.cell.2010.10.002 21094524 PMC3018849

[B101] Starai V. J. Celic I. Cole R. N. Boeke J. D. Escalante-Semerena J. C. (2002). Sir2-dependent activation of acetyl-CoA synthetase by deacetylation of active lysine. Science 298, 2390–2392 10.1126/science.1077650 12493915

[B102] Starai V. J. Escalante-Semerena J. C. (2004). Identification of the protein acetyltransferase (Pat) enzyme that acetylates acetyl-CoA synthetase in *Salmonella enterica*. J. Mol. Biol. 340, 1005–1012 10.1016/j.jmb.2004.05.010 15236963

[B103] Still A. J. Floyd B. J. Hebert A. S. Bingman C. A. Carson J. J. Gunderson D. R. (2013). Quantification of mitochondrial acetylation dynamics highlights prominent sites of metabolic regulation. J. Biol. Chem. 288, 26209–26219 10.1074/jbc.M113.483396 23864654 PMC3764825

[B104] Sundaresan N. R. Gupta M. Kim G. Rajamohan S. B. Isbatan A. Gupta M. P. (2009). Sirt3 blocks the cardiac hypertrophic response by augmenting Foxo3a-dependent antioxidant defense mechanisms in mice. J. Clin. Invest. 119, 2758–2771 19652361 10.1172/JCI39162 PMC2735933

[B105] Sundaresan N. R. Samant S. A. Pillai V. B. Rajamohan S. B. Gupta M. P. (2008). SIRT3 is a stress-responsive deacetylase in cardiomyocytes that protects cells from stress-mediated cell death by deacetylation of Ku70. Mol. Cell. Biol. 28, 6384–6401 10.1128/MCB.00426-08 18710944 PMC2577434

[B106] Tan M. Luo H. Lee S. Jin F. Yang J. S. Montellier E. (2011). Identification of 67 histone marks and histone lysine crotonylation as a new type of histone modification. Cell 146, 1016–1028 10.1016/j.cell.2011.08.008 21925322 PMC3176443

[B107] Tan M. Peng C. Anderson K. A. Chhoy P. Xie Z. Dai L. (2014). Lysine glutarylation is a protein posttranslational modification regulated by SIRT5. Cell Metab. 19, 605–617 10.1016/j.cmet.2014.03.014 24703693 PMC4108075

[B108] Tanner K. G. Landry J. Sternglanz R. Denu J. M. (2000). Silent information regulator 2 family of NAD-dependent histone/protein deacetylases generates a unique product, 1-O-acetyl-ADP-ribose. Proc. Natl. Acad. Sci. U.S.A. 97, 14178–14182 10.1073/pnas.250422697 11106374 PMC18891

[B109] Tao R. Coleman M. C. Pennington J. D. Ozden O. Park S. H. Jiang H. (2010). Sirt3-mediated deacetylation of evolutionarily conserved lysine 122 regulates MnSOD activity in response to stress. Mol. Cell 40, 893–904 10.1016/j.molcel.2010.12.013 21172655 PMC3266626

[B110] Thao S. Escalante-Semerena J. C. (2011). Control of protein function by reversible Nvarepsilon-lysine acetylation in bacteria. Curr. Opin. Microbiol. 14, 200–204 10.1016/j.mib.2010.12.013 21239213 PMC3078959

[B111] Thauer R. K. Jungermann K. Decker K. (1977). Energy conservation in chemotrophic anaerobic bacteria. Bacteriol. Rev. 41, 100–180 860983 10.1128/br.41.1.100-180.1977 PMC413997

[B112] Tweedie-Cullen R. Y. Brunner A. M. Grossmann J. Mohanna S. Sichau D. Nanni P. (2012). Identification of combinatorial patterns of post-translational modifications on individual histones in the mouse brain. PLoS ONE 7:e36980 10.1371/journal.pone.0036980 22693562 PMC3365036

[B113] Vadvalkar S. S. Baily C. N. Matsuzaki S. West M. Tesiram Y. A. Humphries K. M. (2013). Metabolic inflexibility and protein lysine acetylation in heart mitochondria of a chronic model of type 1 diabetes. Biochem. J. 449, 253–261 10.1042/BJ20121038 23030792 PMC3518897

[B114] Wagner G. R. Payne R. M. (2013). Widespread and enzyme-independent Nepsilon-acetylation and Nepsilon-succinylation of proteins in the chemical conditions of the mitochondrial matrix. J. Biol. Chem. 288, 29036–29045 10.1074/jbc.M113.486753 23946487 PMC3790002

[B115] Wagner G. R. Pride P. M. Babbey C. M. Payne R. M. (2012). Friedreich's ataxia reveals a mechanism for coordinate regulation of oxidative metabolism via feedback inhibition of the SIRT3 deacetylase. Hum. Mol. Genet. 21, 2688–2697 10.1093/hmg/dds095 22394676 PMC3363336

[B116] Wang Q. Zhang Y. Yang C. Xiong H. Lin Y. Yao J. (2010). Acetylation of metabolic enzymes coordinates carbon source utilization and metabolic flux. Science 327, 1004–1007 10.1126/science.1179687 20167787 PMC4183141

[B117] Waterson R. M. Hill R. L. (1972). Enoyl coenzyme A hydratase (crotonase). Catalytic properties of crotonase and its possible regulatory role in fatty acid oxidation. J. Biol. Chem. 247, 5258–5265 5057465

[B118] Weinert B. T. Iesmantavicius V. Moustafa T. Scholz C. Wagner S. A. Magnes C. (2013b). Acetylation dynamics and stoichiometry in *Saccharomyces cerevisiae*. Mol. Syst. Biol. 10, 716 10.1002/msb.134766 24489116 PMC4023402

[B119] Weinert B. T. Iesmantavicius V. Wagner S. A. Scholz C. Gummesson B. Beli P. (2013a). Acetyl-phosphate is a critical determinant of lysine acetylation in *E. coli*. Mol. Cell 51, 265–272 10.1016/j.molcel.2013.06.003 23830618

[B120] Weinert B. T. Scholz C. Wagner S. A. Iesmantavicius V. Su D. Daniel J. A. (2013c). Lysine succinylation is a frequently occurring modification in prokaryotes and eukaryotes and extensively overlaps with acetylation. Cell Rep. 4, 842–851 10.1016/j.celrep.2013.07.024 23954790

[B121] Weinert B. T. Wagner S. A. Horn H. Henriksen P. Liu W. R. Olsen J. V. (2011). Proteome-wide mapping of the Drosophila acetylome demonstrates a high degree of conservation of lysine acetylation. Sci. Signal. 4, ra48 10.1126/scisignal.2001902 21791702

[B122] Xie Z. Dai J. Dai L. Tan M. Cheng Z. Wu Y. (2012). Lysine succinylation and lysine malonylation in histones. Mol. Cell. Proteomics 11, 100–107 10.1074/mcp.M111.015875 22389435 PMC3418837

[B123] Yang Y. Cimen H. Han M. J. Shi T. Deng J. H. Koc H. (2010). NAD+-dependent deacetylase SIRT3 regulates mitochondrial protein synthesis by deacetylation of the ribosomal protein MRPL10. J. Biol. Chem. 285, 7417–7429 10.1074/jbc.M109.053421 20042612 PMC2844190

[B124] Yoshino J. Mills K. F. Yoon M. J. Imai S. (2011). Nicotinamide mononucleotide, a key NAD(+) intermediate, treats the pathophysiology of diet- and age-induced diabetes in mice. Cell Metab. 14, 528–536 10.1016/j.cmet.2011.08.014 21982712 PMC3204926

[B125] Yu B. J. Kim J. A. Moon J. H. Ryu S. E. Pan J. G. (2008). The diversity of lysine-acetylated proteins in *Escherichia coli*. J. Microbiol. Biotechnol. 18, 1529–1536 18852508

[B126] Yu J. Sadhukhan S. Noriega L. G. Moullan N. He B. Weiss R. S. (2013). Metabolic characterization of a Sirt5 deficient mouse model. Sci. Rep. 3:2806 10.1038/srep02806 24076663 PMC3786297

[B127] Yu W. Dittenhafer-Reed K. E. Denu J. M. (2012). SIRT3 protein deacetylates isocitrate dehydrogenase 2 (IDH2) and regulates mitochondrial redox status. J. Biol. Chem. 287, 14078–14086 10.1074/jbc.M112.355206 22416140 PMC3340192

[B128] Yu W. Lin Y. Yao J. Huang W. Lei Q. Xiong Y. (2009). Lysine 88 acetylation negatively regulates ornithine carbamoyltransferase activity in response to nutrient signals. J. Biol. Chem. 284, 13669–13675 10.1074/jbc.M901921200 19318352 PMC2679468

[B129] Zhang J. Sprung R. Pei J. Tan X. Kim S. Zhu H. (2009). Lysine acetylation is a highly abundant and evolutionarily conserved modification in *Escherichia coli*. Mol. Cell. Proteomics 8, 215–225 10.1074/mcp.M800187-MCP200 18723842 PMC2634580

[B130] Zhang K. Zheng S. Yang J. S. Chen Y. Cheng Z. (2013). Comprehensive profiling of protein lysine acetylation in *Escherichia coli*. J. Proteome Res. 12, 844–851 10.1021/pr300912q 23294111

[B131] Zhang Z. Tan M. Xie Z. Dai L. Chen Y. Zhao Y. (2011). Identification of lysine succinylation as a new post-translational modification. Nat. Chem. Biol. 7, 58–63 10.1038/nchembio.495 21151122 PMC3065206

[B132] Zhao K. Chai X. Marmorstein R. (2004). Structure and substrate binding properties of cobB, a Sir2 homolog protein deacetylase from *Escherichia coli*. J. Mol. Biol. 337, 731–741 10.1016/j.jmb.2004.01.060 15019790

[B133] Zhao S. Xu W. Jiang W. Yu W. Lin Y. Zhang T. (2010). Regulation of cellular metabolism by protein lysine acetylation. Science 327, 1000–1004 10.1126/science.1179689 20167786 PMC3232675

[B134] Zhu W. Z. Wu X. F. Zhang Y. Zhou Z. N. (2012). Proteomic analysis of mitochondrial proteins in cardiomyocytes from rats subjected to intermittent hypoxia. Eur. J. Appl. Physiol. 112, 1037–1046 10.1007/s00421-011-2050-9 21735218

